# Prevalence of canine distemper in minks, foxes and raccoon dogs from 1983 to 2023 in Asia, North America, South America and Europe

**DOI:** 10.3389/fvets.2024.1394631

**Published:** 2024-08-13

**Authors:** Jian Liang, Tingting Wang, Qi Wang, Xiaolin Wang, Xinying Fan, Tingting Hu, Xue Leng, Kun Shi, Jianming Li, Qinglong Gong, Rui Du

**Affiliations:** ^1^College of Animal Science and Technology, Jilin Agricultural University, Changchun, Jilin, China; ^2^College of Chinese Medicine Materials, Jilin Agricultural University, Changchun, Jilin, China; ^3^Laboratory of Production and Product Application of Sika Deer of Jilin Province, Jilin Agricultural University, Changchun, Jilin, China

**Keywords:** minks, foxes, raccoon dogs, canine distemper, Meta

## Abstract

Canine distemper (CD) is a virulent disease caused by the canine distemper virus (CDV) in canines and mustelidaes with high mortality. The incidence of CDV is worldwide distribution and it has caused huge economic losses to multiple industries around the world. There are many studies investigating the prevalence of CD infection, but no comprehensive analysis of CDV infection in minks, foxes and raccoon dogs worldwide has therefore been carried out. The aim of this meta is to provide a comprehensive assessment of the prevalence of CDV infection in minks, foxes and raccoon dogs dogs through a meta-analysis of articles published from around the world. Data from 8,582 small carnivores in 12 countries were used to calculate the combined prevalence of CD. A total of 22.6% (1,937/8,582) of minks, foxes and raccoon dogs tested positive for CD. The prevalence was higher in Asia (13.8, 95% CI: 22.2–45.6), especially in South Korea (65.8, 95% CI: 83.3–95.8). Our study found that the incidence of CD was also associated with geographic climate, population size, health status, and breeding patterns. CD is more commonly transmitted in minks, foxes and raccoon dogs. However, the concentrated breeding as an economic animal has led to an increase in the prevalence rate. The difference analysis study recommended that countries develop appropriate preventive and control measures based on the prevalence in the minks, foxes, and raccoon dogs industries, and that reducing stocking density is important to reduce the incidence of CDV. In addition, CDV is more common in winter, so vaccination in winter should be strengthened and expanded to reduce the incidence of CD in minks, foxes and raccoon dogs.

## 1 Introduction

Canine distemper virus (CDV) is an enveloped, negative, single-stranded RNA virus belonging to the genus Measles virus, a member of the family Paramyxoviridae (1). Other members of the genus, such as rinderpest virus (RPV) and measles virus (MV), are known to cause devastating diseases in animals (2). CDV is capable of infecting a wide range of species and poses a serious threat to the conservation of animals. The disease was first reported in Spain (1,761) and is thought to have spread from there to the rest of the world (3). Canine distemper has long been a serious and fatal disease of a large number of carnivores, and poses a significant threat to the health of small carnivores such as minks, foxes and raccoon dogs in particular (4). CDV is similar to other paramyxoviruses in that the virus contains six structural proteins, nucleocapsid (N), phosphoprotein (P), large (L), matrix (M), hemagglutinin (H), and fusion (F) proteins, as well as two auxiliary Non-structural proteins (C and V), which are present as extra-transcriptional units of the P gene (5). Mutations affecting the CDV H protein required for viral attachment to host cell receptors have been associated with virulence and disease emergence in novel host species.

The virus is mainly transmitted directly or indirectly through aerosols and contact with respiratory and ocular secretions (6). Other body excretions and secretions (e.g., urine and feces) may contribute to the transmission of the virus during the acute phase of infection above. Generally, CDV exhibits lymphatic, neurological, and epithelial characteristics, resulting in systemic infections of almost all organ systems including respiratory, digestive, urinary, lymphatic, endocrine, cutaneous, skeletal and central nervous system (CNS) (7, 8). The disease course and pathogenesis in CD resemble those of human measles virus infection including, fever, rash, respiratory signs, lymphopenia, and severe immunosuppression with generalized depletion of lymphatic organs during the acute disease phase (7). In addition, CDV infection shows a high incidence of neurological complications (8). Initially, the natural hosts of CDV were canids but as wildlife habitats have changed and the virus itself has evolved, the natural hosts of CDV have expanded to include Felidae, Viverridae, Mustelidae, Mephitidae, Ursidae, Procyonidae, Ailuride and Huaenidae, among others (9). In total, more than 20 carnivorous and non-carnivorous families have been reported to be affected (10).

Economically, minks, foxes and raccoon dogs play an indispensable role in the breeding industry of various countries as fur animals. For example, according to the International Fur Association, Northern Europe is the largest mink breeding region in the world, with around 58% of the total, while other major breeding regions include China, North America, Russia, Argentina and Ukraine (11). Denmark is currently the world’s largest martens producer and furexports are an important pillar of the Danish industry (12). The increasing demand for minks, foxes, and raccoon dogs farming is gradually expanding. CDV is expanding host range and high mortality rates seriously threaten the sustainability of the minks, foxes and raccoon dogs farming industry (13). Recent studies have shown that lethal infections also occur in non-carnivorous species such as wild boar and non-human primates, suggesting that the pathogen has a remarkable ability to cross species barriers (14).

Epidemiological data from around the world indicate that CDV has become a major threat to many protected species, even those outside the order Carnivora (15). The importance of infection in multi-host situations is not fully understood due to the lack of epidemiologic information on CDV transmission. Understanding the epidemiology of CDV is important not only for preventive diagnosis of minks, foxes and raccoons, but also for the development of reliable wildlife conservation strategies. However, to the best of our knowledge, there is currently no adequate systematic analysis of the overall prevalence rate of CD infection in minks, foxes, and raccoon dogs in the world, so we conducted a study to estimate the prevalence of CDV infection in mink, fox, and raccoon dog populations globally and to assess potential risk factors associated with the prevalence rate of CD disease. This study will help to understand the epidemiology of CDV in minks, foxes and raccoon dogs and improve timely prevention of CDV-induced diseases in minks, foxes and raccoon dogs.

## 2 Materials and methods

### 2.1 Search strategy and selection criteria

PRISMA is used to report the results of our systematic review and meta-analysis. We searched for papers published from 1983 to December 8, 2022 in VIP Chinese Journal Database, CNKI, Wanfang Database, PubMed and ScienceDirect, Web of Science. Our aim was to screen all papers published in English or Chinese on the world epidemic of Canine distemper. We attempted to contact the authors of studies that could not be downloaded from the database for additional information. In the PubMed database, we used the MeSH terms “mink,” “fox,” “raccoon dog,” and “Canine distemper” to retrieve the medical subject terms and their free words associated with them. The subject terms were linked using the Boolean operator “AND” and the free terms were linked using the Boolean operator “OR” to generate the final search formula.

Total: ((((((((((((((Minks) OR (*Mustela vison*)) OR (American Mink)) OR (Mink, American)) OR (*Mustela macrodon*)) OR (Sea Mink)) OR (Mink, Sea)) OR (Minks, Sea)) OR (Sea Minks)) OR (*Mustela lutreola*)) OR (European Mink)) OR (Mink, European)) AND (((Canine Distemper Virus) OR (Canine Distemper Viruses)) OR (Distemper Viruses, Canine))) OR ((((Canine Distemper Virus) OR (Canine Distemper Viruses)) OR (Distemper Viruses, Canine)) AND (((((((((Vulpes) OR (Pseudalopex)) OR (Urocyon)) OR (*Vulpes vulpes*)) OR (Red Fox)) OR (Fox, Red)) OR (Alopex)) OR (Arctic Fox)) OR (Fox, Arctic)))) OR ((((((Dog, Raccoon) OR (Dogs, Raccoon)) OR (Raccoon Dog)) OR (*Nyctereutes procyonoides*)) OR (Nyctereutes)) AND (((Canine Distemper Virus) OR (Canine Distemper Viruses)) OR (Distemper Viruses, Canine))).

In the Web of Science, we use the terms “mink,” “fox,” “raccoon dog,” and “Canine distemper “. The Boolean operators “AND” and “OR” are used to link medical terms in the advanced search.

Search in ScienceDirect using keywords such as “mink,” “fox,” “raccoon dog,” “Canine distemper,” and “research article type.” In the advanced search of three Chinese databases (CNKI, Wanfang and VIP databases), the same Chinese search terms are also used, including fuzzy search and synonym expansion.

All retrieved citations are imported into Endnote X9 (9.3.3). Eligible studies were screened according to the following criteria:

1. Study subjects must be “minks,” “foxes” and “raccoon dogs”.
2. The aim of the study must be to investigate the positive rate of Canine distemper infection in minks, foxes and raccoon dogs.
3. The data must include the numbers of minks, foxes and raccoon dogs.
4. The research design must be a cross-sectional study.
5. Research must be published in Chinese or English.

Studies that did not meet all of the above criteria were excluded. Repeated studies and review studies (non-research papers) were also excluded.

### 2.2 Selection criteria

The reviewers extracted the following variables from each study separately: year of sampling, first author, year of publication, country, region, assay method, sample type, the season of collection, population size, sex and age of the sampled animals, breeding pattern, type of animals sampled, health status, data of geographical factors (longitude, latitude, mean annual rainfall, altitude, mean annual temperature, mean annual humidity) taken from the National Meteorological Information Center of the China Meteorological Administration. The primary reviewer (QLG) confirms all extracted data. The ‘quality’ of each included study was assessed by using criteria derived from the GRADE (Grading of Recommendations for Assessment, Development and Evaluation) methodology. The scoring method are used for grading, and each of the criteria mentioned below identified as 1 point: (i) random sampling; (ii) a clear method of detection; (iii) provision of a detailed description of the sampling method; (iv) a clear sampling time; and (v) include four or more risk factors.

### 2.3 Statistical analysis

We performed meta-analysis using the “meta” package in R software (“R Core Team, Version 4.0.0; “R: Language and Environment for Statistical Computing,” R Core Team, 2022) (16). We use the Freeman-Tukey double anti-sine transform (Named “PFT” in the tuple) for transform to conform to the normal distribution (17). The composite estimates included in the study were described using forest plots. Heterogeneity of prevalence meta-analyses is usually large, so we made judgments in advance and used random effects models to analyze overall prevalence (including subgroups). Differences due to the heterogeneity of the included studies were evaluated using Cochrane Q-statistics and Higgin statistics. In a funnel plot, the symmetry of the graph is judged subjectively. If the points in a funnel plot are symmetrically distributed on either side of the line of symmetry, there is no publication bias, and if they are not symmetrical, there is publication bias in the included studies. At the same time in order to trace potential sources of heterogeneity present in our study, we conducted subgroup analyses and univariate meta-regression. Potential factors include geographical region (Asia, Europe, South America, North America); sampling of years (before 1988 and 2008 or later); Detection methods (serology, molecular biology); feeding pattern (intensive, free-range); age (≤1 year, >1 year); sex (male, female); season (spring, summer, autumn, winter), rating level (2–3 points, 4–5 points). Using the data from the National Meteorological Information Center of the China Meteorological Administration, geographical factors were further extracted based on sampling location using subgroup analysis and one-way meta-regression analysis to trace the sources of heterogeneity. The R software codes for this study is shown in Table S2.

## 3 Results

### 3.1 Search results and eligible studies

**Figure 1:**
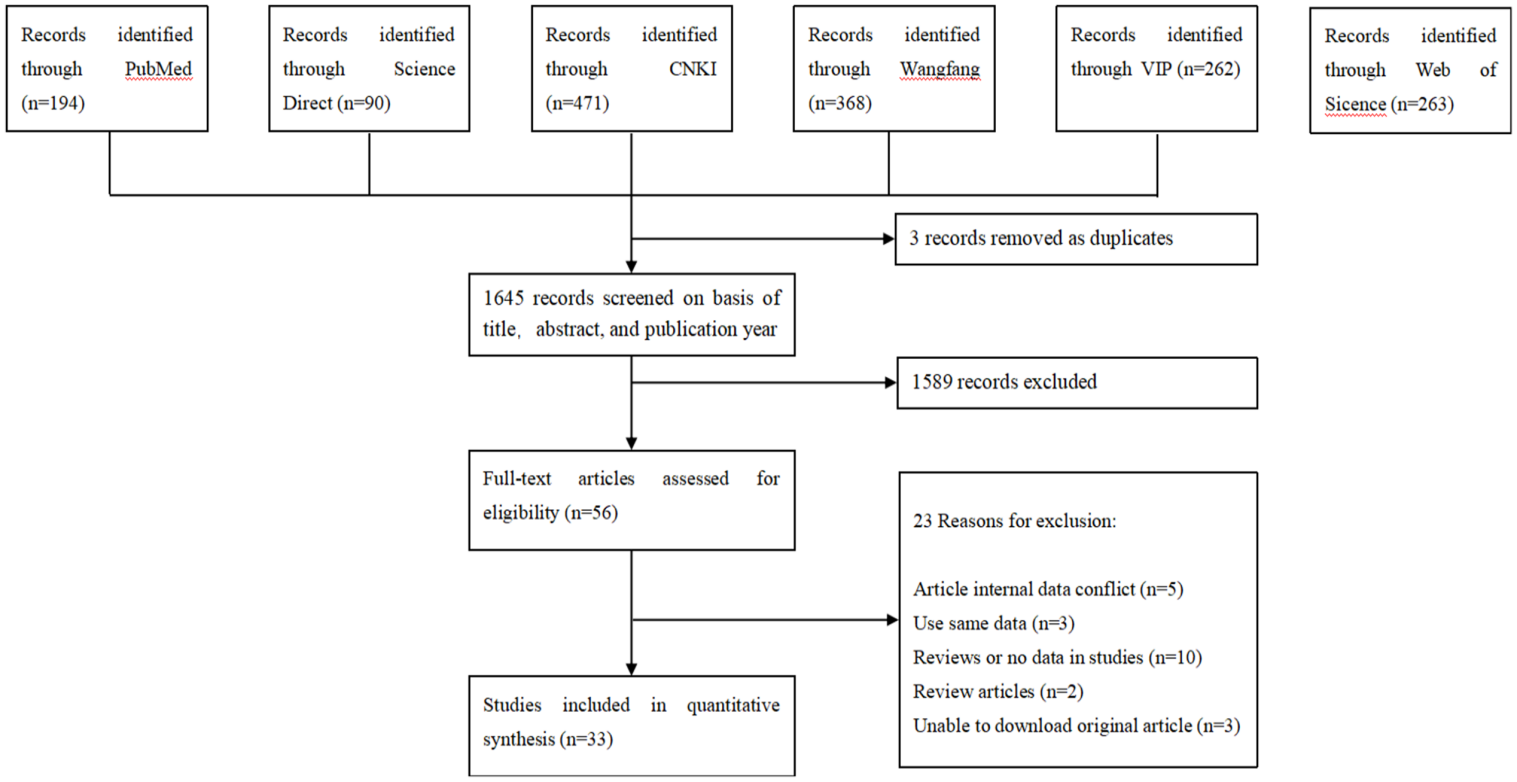
Flow diagram of eligible studies for searching and selecting.

According to our inclusion criteria, 1,648 articles were collected from six databases, and 33 studies were finally included to establish this meta-analysis (Figure 1). A total of 9 studies were divided into 4–5 points, and 24 studies were divided into 2–3 points (Tables S2, S3).

### 3.2 Publication bias and sensitivity analysis

**Figure 2:**
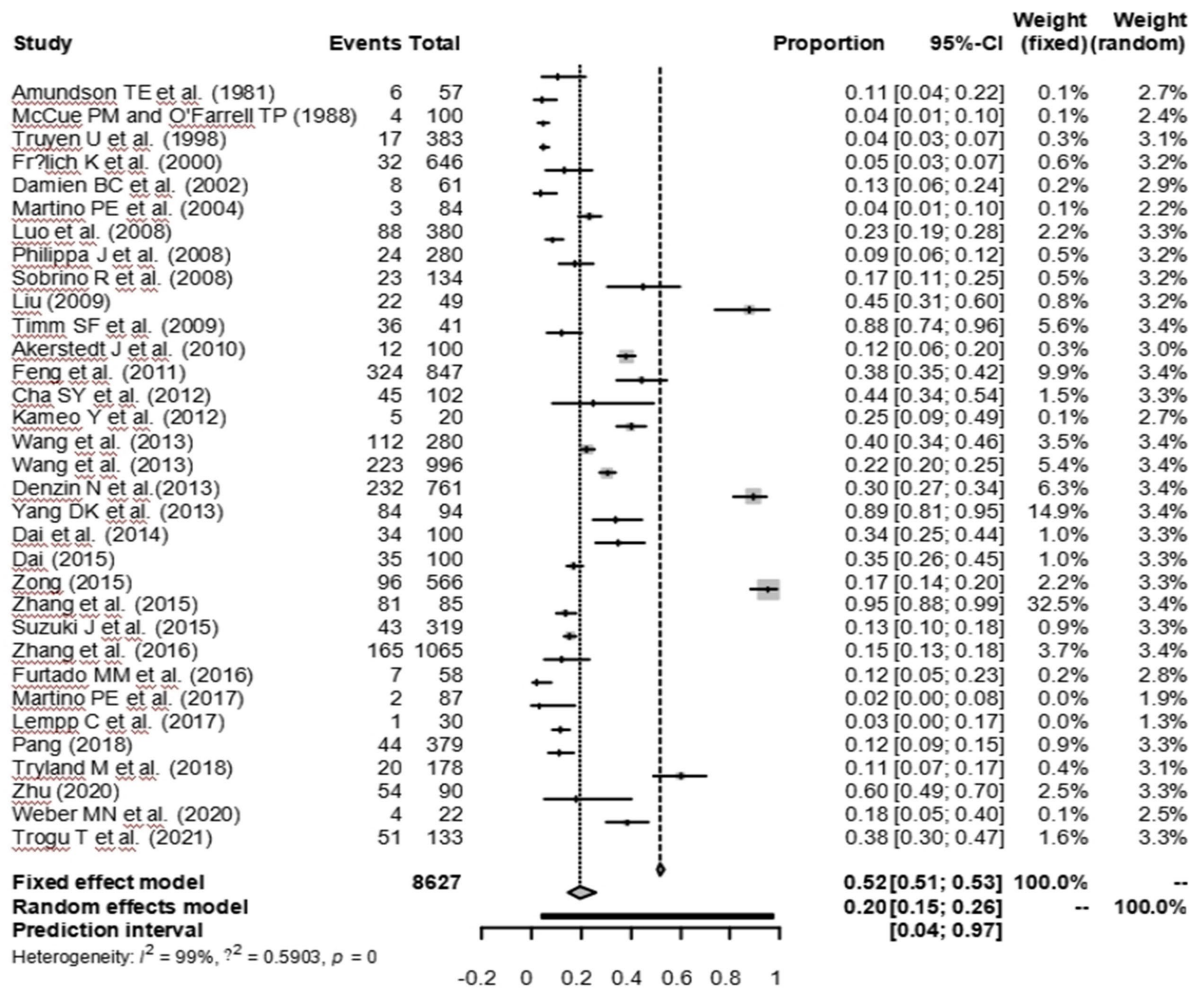
Forest plot of prevalence of Canine distemper in minks, foxes and raccoon dogs among studies conducted in the World.

**Figure 3:**
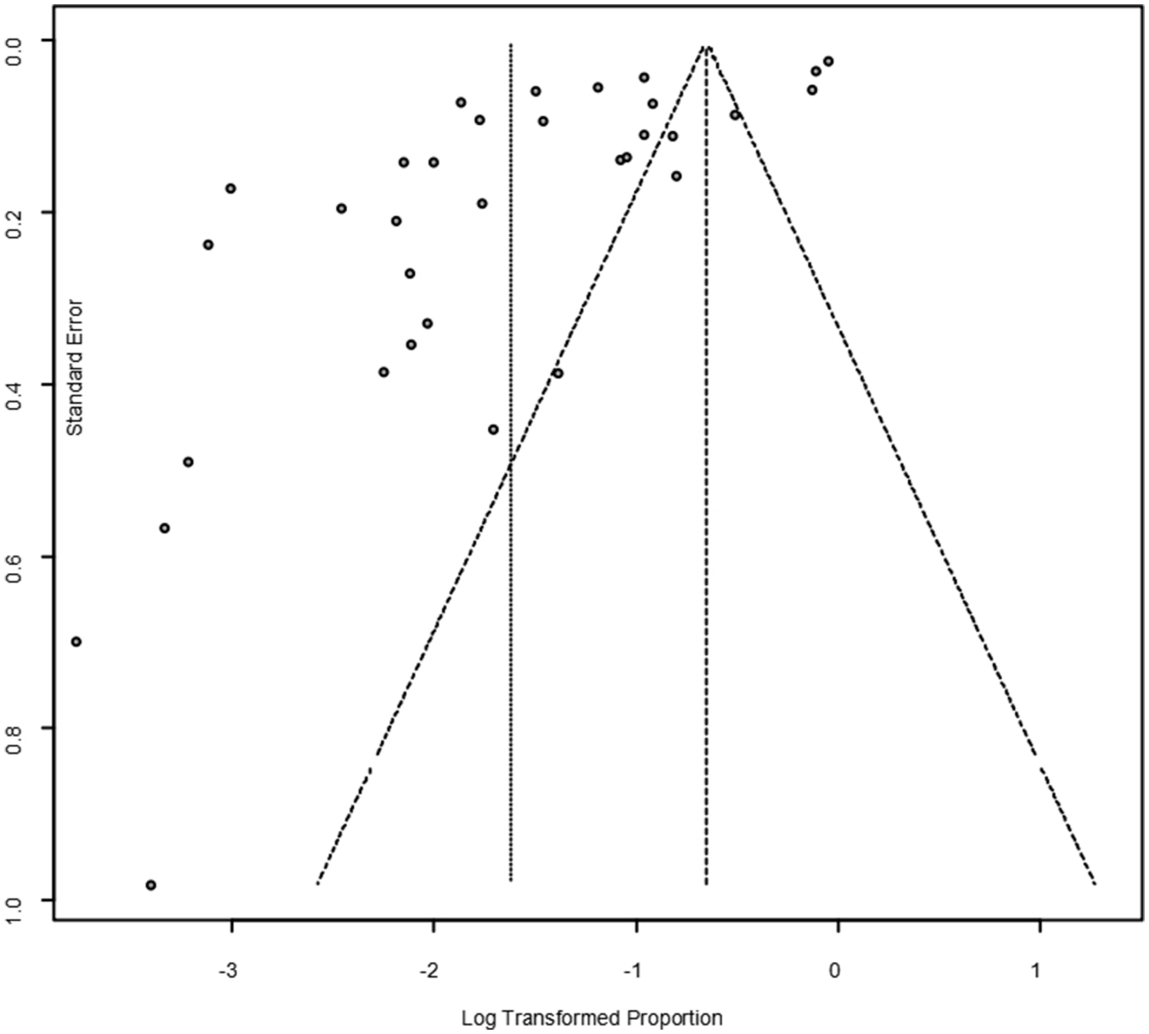
Funnel plot with pseudo 95% confidence interval limits for the examination of publication bias.

**Figure 4:**
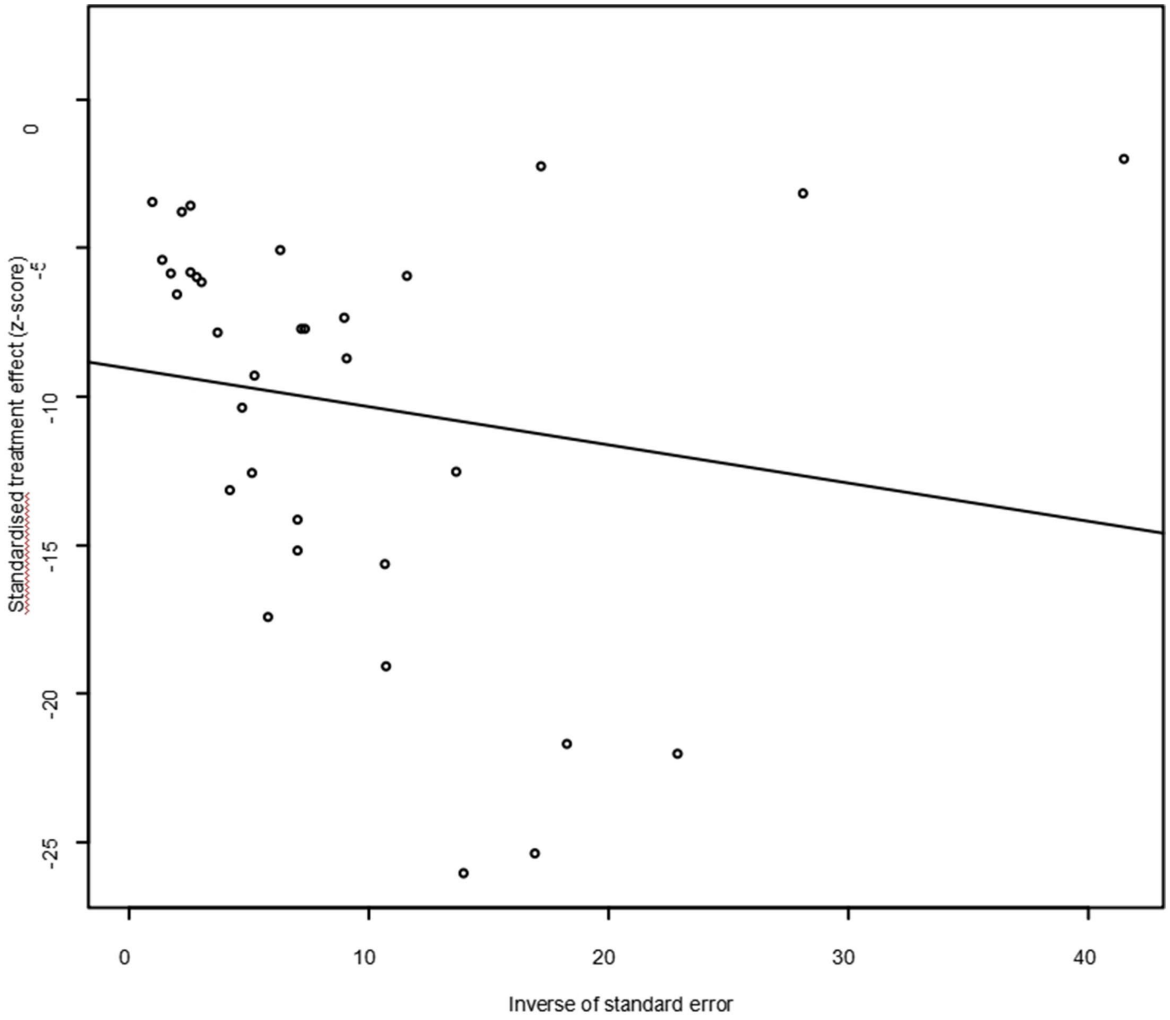
Egger’s test for publication bias.

As suggested by previous studies, we finally chose PFT to perform rate conversion (Table 1). The forest plots. Results showed a high heterogeneity in the included studies (I2 = 99.8%, *p* < 0.01; Figure 2). In the funnel plot, we observed asymmetry, indicating publication bias in our meta-analysis (Figure 3). The results of the egger test were the same as for the funnel plot (*t* = −5.216, *p* = 0.05; Figure 4; Supplementary Table S4). Pruning and padding analyses were shown to indicate publication bias or small sample bias in our included studies (Supplementary Figure S2). Sensitivity analyses verified the reliability of the results, and the exclusion of any one study had little effect on the overall quality of the meta-analysis (Supplementary Figure S1). We also provide funnel plots for each subgroup to determine whether publication bias or small sample bias was present (Supplementary Figures S3–S10).

**Table 1:** Normal distribution test for the normal rate and the different conversion of the normal rate.

Conversion form	W	P
PRAW	0.81465	6.218e-05
PLN	0.96803	0.4278
PLOGIT	0.92927	0.03336
PAS	0.87633	0.001369
PFT	0.86896	0.0009163

“PRAW”: original rate; “PLN”: logarithmic conversion; “PLOGIT”: logit transformation; “PAS”: arcsine transformation; “PFT”: double-arcsine transformation; “NaN”: meaningless number; “NA”: missing data.

### 3.3 Meta-analysis

**Figure 5:**
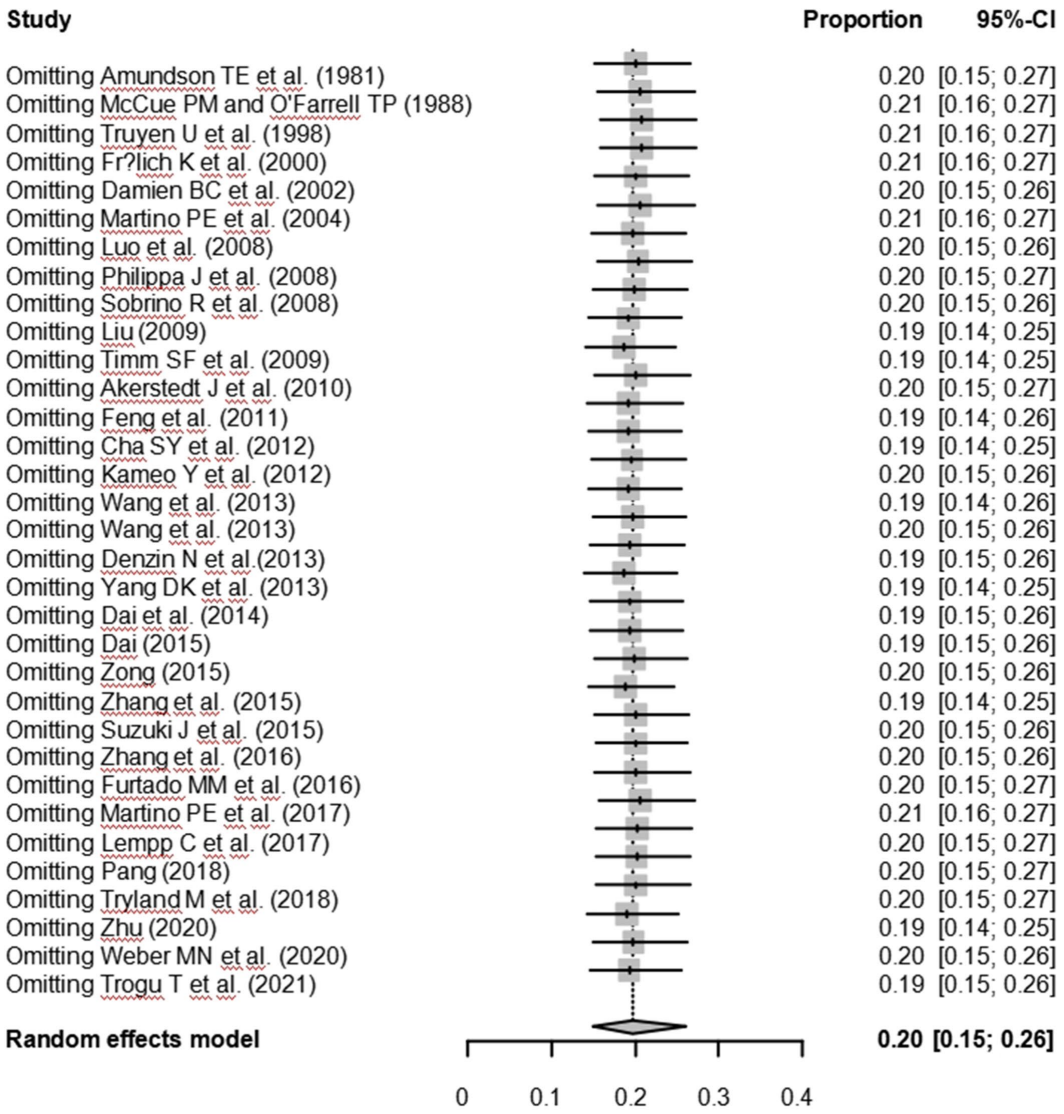
Sensitivity analysis.

The combined prevalence of CD infection was 22.6% (95% CI: 0.1–0.3; 1,937/8,582) of the 33 studies selected (Table 2). Regionally, North America had the highest prevalence 16.1% (95% CI: 2.1–100; 46/198), while South America had the lowest prevalence (7.3%; 95% CI: 3.0–17.6; 16/251), and all other continents had a prevalence of more than 10% (Table 2). In the sampling year subgroup, we found that the prevalence of CD in minks, foxes and raccoon dogs were on the rise, with the highest infection rate of 25.9% (95% CI: 17.1–39.3) after 2010. In terms of species, the prevalence was highest in raccoon dogs (36.1%; 95% CI: 19.4–67.2), lowest in foxes (18.9%; 95% CI: 13.1–27.3), and 23.2% in minks (95% CI: 17.1–31.8); in terms of age, the prevalence was 19.0% (95% CI: 8.2–44.2) in animals sampled >6 months old, which was lower than that of animals <6 months old 20.1% (95% CI: 11.8–34.3); in the subgroup of detection methods, the estimated pooled prevalence of CD using serological detection was 16.9% (95% CI: 11.9–24.1) lower than that using molecular biology 27.1% (95% CI: 21.4–34.1). In season, CD disease was more prevalent in winter 24.2% (95% CI: 14.1–41.7). In the feeding mode subgroup, the prevalence of breeding mode was 30.6% (95% CI: 18.1–51.9), which was significantly higher than that of free-range mode 6.9% (95% CI: 4.9–9.8) and wild minks, foxes and raccoon dogs 24.1% (95% CI: 12.6–46.2). In the population size subgroup, the prevalence of animals >500 (9.8%; 95% CI: 6.4–14.9) was lower than that of animals <500 (17%; 95% CI: 12.9–22.3). Prevalence was higher in articles scoring 2–3 (23.5%; 95% CI: 17.2–32.1). We also conducted subgroup analyses for geographical factors. The highest prevalence was observed at longitudes of 120°E and above 32.0% (95% CI: 21.1–48.5), as well as at latitudes of 30°-60°N 25.0% (95% CI: 17.1–36.6; Figure 5).

**Table 2:** Studies included in the analysis.

Reference ID	Sampling time	Country	Detection method	No. tested	No. positive	Prevalence	Quality level
Asia							
Luo et al. (2008)	2006–2007	China	Molecular Biology	522	10	0.019157088	middle
Liu (2009)	2009–2015	China	Molecular Biology	553	236	0.426763110	middle
Feng et al. (2011)	2009–2010	China	Molecular Biology	107	7	0.065420561	middle
Kameo et al. (2012)	2007–2008	Japan	Serology	502	30	0.059760956	middle
Cha et al. (2012)	2010–2011	Korea	Molecular Biology	91	22	0.241758242	middle
Wang et al. (2013a)	2010–2012	China	Molecular Biology	642	149	0.232087227	middle
Wang et al. (2013b)	2011–2012	China	Serology	90	24	0.266666667	middle
Yang et al. (2013)	2011–2012	Korea	Serology	156	30	0.192307692	middle
Dai et al. (2014)	2013	China	Serology	150	12	0.080000000	high
Dai (2015)	2013	China	Serology	214	21	0.098130841	high
Zong (2015)	2014	China	Molecular Biology	321	19	0.059190031	middle
Suzuki et al. (2015)	2006–2012	Japan	Serology	368	62	0.168478261	middle
Zhang et al. (2015)	UN	China	Serology	300	6	0.020000000	middle
Zhang et al. (2016)	2011–2013	China	Serology	47	4	0.085106383	middle
Pang (2018)	UN	China	Molecular Biology	1,248	39	0.031250000	middle
Zhu (2020)	UN	China	Serology	40	6	0.150000000	middle
Europe							
Truyen et al. (1998)	1991–1995	Germany	Serology	383	17	0.044386423	middle
Frölich et al. (2000)	1996–1998	Germany	Serology	601	32	0.053244592	middle
Damien et al. (2002)	1997–1997	Luxembourg	Serology	61	8	0.131147541	high
Philippa et al. (2008)	1996–2003	France	Serology	280	24	0.085714286	high
Sobrino et al. (2008)	1997–2007	Spanish	Serology	134	23	0.171641791	middle
Akerstedt et al. (2010)	1994–2005	Norway	Serology	100	12	0.12	middle
Denzin et al.(2013)	2010–2011	Germany	Molecular Biology	761	232	0.304862024	middle
Lempp et al. (2017)	2013–2016	Germany	Serology	30	1	0.033333333	high
Tryland et al. (2018)	1995–2003	Norway	Serology	178	20	0.112359551	middle
Trogu et al. (2021)	2018–2020	Italy	Molecular Biology	133	51	0.383458647	middle
North America							
Amundson et al. (1981)	1978–1979	USA	Serology	57	6	0.105263158	high
McCue PM and O’Farrell TP (1988)	1981–1984	USA	Serology	100	4	0.04	high
Timm et al. (2009)	1999–2000	USA	Serology	41	36	0.87804878	middle
South America							
Martino et al. (2004)	1998–2001	Argentina	Serology	84	3	0.035714286	high
Furtado et al. (2016)	2000–2008	Brazil	Serology	58	7	0.120689655	high
Martino et al. (2017)	2013–2015	Argentina	Serology	87	2	0.022988506	middle
Weber et al. (2020)	2017–2019	Brazil	Molecular Biology	22	4	0.181818182	middle

Quality*: low (0–1), medium (2–3), high (4–5).

## 4 Discussion

Infections caused by Canine distemper virus (CDV) are highly lethal to minks, foxes and raccoon dogs and have a significant impact on the fur animal farming industry (14). Therefore, we conducted a meta-analysis of Canine distemper virus infections in minks, foxes and raccoon dogs around the world, using stress sampling years and other methods (Table 3).

**Table 3:** Pooled prevalence of canine distemper virus of small carnivores in the world.

		No. studies	No. tested	No. positive	% (95% CI*)	Heterogeneity			Univariate meta-regression	
						χ^2^	*p*-value	I^2^ (%)	*p*-value	Coefficient (95% CI)
Region*										
	Asia	16	5,472	1,455	13.8% (22.2–45.6)	1384.22	0.00	99.2%	0.0004	1.004 (0.4472 to 1.5609)
	Europe	10	2,661	420	11.2% (7.0–19.8)	227.47	< 0.01	96.0%		
	South America	4	251	16	7.3% (3.0–17.6)	9.577	0.02	68.7%		
	North America	3	198	46	16.1% (2.1–100)	67.53	< 0.01	97.0%		
Sampling yeas										
	before 2000	7	1,421	123	10.3% (2.8–38.2)	486.25	< 0.01	98.8%	0.0189	−0.8412 (−1.5434 to −0.1390)
	2000 to 2010	41	1,305	424	24.7% (16–38.0)	33.26	< 0.01	91.0%		
	2010 or late	13	4,336	1,804	25.9% (17.1–39.3)	919.46	< 0.01	98.7%		
Species										
	Mink	17	4,205	820	23.2% (17.1–31.8)	336.48	< 0.01	95.2%		
	Fox	20	3,722	893	18.9% (13.1–27.3)	1281.28	< 0.01	98.5%		
	Racconn Dog	7	655	224	36.1% (19.4–67.2)	231.02	< 0.01	97.4%	0.1239	0.5549 (−0.1520 to 1.2618)
Detection method*										
	Serology	23	4,347	808	16.9% (11.9–24.1)	1748.73	0.00	98.7%		
	Molecular biology	10	4,235	1,129	27.1% (21.4–34.1)	169.97	< 0.01	94.7%	0.1193	0.4259 (−0.1100 to 0.9619)
Sample										
	Seurm	20	2,896	555	18.5% (13.2–25.9)	1168.00	< 0.01	98.4%		
	Tissue	12	4,621	1.217	24.2% (19.5–30.7)	200.15	< 0.01	94.5%	0.3947	0.2217 (−0.289 to 0.7322)
	Secretion	1	1,065	165	19.6% (13.5–17.8)	0.00	un	–		
Breeding mode										
	Intensive	9	4,419	1,158	30.6%(18.1–51.9)	1419.50	< 0.01	99.4%	0.0229	0.8844 (0.1228 to 1.6461)
	Free-ranging	8	1,650	103	6.9% (4.9–9.8)	20.01	< 0.01	65.0%		
	Wild	8	898	253	24.1% (12.6–46.2)	328.92	< 0.01	97.9%		
Longitude										
	Less90W	4	509	36	6.8% (3.8–12.2)	6.68	0.08	55.1%	0.0035	−1.3807 (−2.3080 to −0.4535)
	Less90E	3	632	75	14.0% (3.0–66.1)	113.45	< 0.01	98.2%		
	90-120 W	1	100	4	4.0% (1.5–10.5)	0.00	un	–		
	90-120E	4	1,469	297	25.4% (7.4–87.0)	723.84	< 0.01	99.6%		
	more120E	10	2,709	833	32.0% (21.1–48.5)	626.90	< 0.01	98.6%		
Latitude										
	0-30S	1	58	7	12.1% (6.0–24.2)	0.00	un			
	30-60S	2	171	5	3.0% (1.3–7.1)	0.24	0.62	0.0%	0.0061	1.3237 (0.3783 to 2.2692)
	30-60 N	16	5,012	1,213	25.0% (17.1–36.6)	1775.87	0.00	99.2%		
	60-90 N	1	178	20	11.2% (7.4–17.0)	0.00	un			
Season*										
	Spring	4	377	107	17.7% (8.3–37.8)	20.03	< 0.01	85.0%		
	Summer	6	1,454	286	16.3% (10.4–25.5)	68.81	< 0.01	92.7%	0.2629	−0.3520 (−0.9682 to 0.2642)
	Autumn	6	1,295	301	23.7% (16.5–34.2)	68.82	< 0.01	92.7%		
	Winter	7	649	226	24.2% (14.1–41.7)	162.48	< 0.01	96.3%		
Age*										
	Juvenile	5	2,151	569	20.1% (11.8–34.3)	140.10	< 0.01	97.1%	0.8135	−0.1128 (−1.0493 to 0.8238)
	Adults	6	638	186	19.0% (8.2–44.2)	247.73	< 0.01	98.0%		
Health										
	Good	6	3,187	807	27.2% (18.8–39.5)	182.83	< 0.01	97.3%	< 0.01	1.8986 (0.9953 to 2.8019)
	Dead	1	601	32	5.0% (3.5–6.9)	0.00	un	–		
	Sick	1	87	2	2.3% (0.6–9.1)	0.00	un	–		
Population										
	<500	3	832	148	17% (12.9–22.3)	5.41	0.07	63.0	0.0296	0.5480 (0.0541 to 1.0419)
	>500	3	394	37	9.8% (6.4–14.9)	2.75	0.25	27.3%		
Quality level										
	Middle	24	7,712	1,815	23.5% (17.-32.1)	2462.80	0.00	99.1%	0.0279	0.7162 (0.0778 to 1.3545)
	High	9	870	122	11.4% (6.6–20.1)	79.49	< 0.01	89.9%%		
Total		33	8,582	1,937	22.6% (15.0–26.0)	2679.02	0.000	98.8%		

CI*: Confidence interval; Region*: Asia (China, Japan, Korea), Europe (Norway, Luxembourg. Germany, France, Spanish, Italy), North America (the United State), South America (Argentina, Brazil). Method*: Serology: Colloidal Gold; ELISA (Enzyme Linked Immunosorbent Assay); VNT (Virus Neutralization Test); SNT (Serum Neutralization Test); IFA (Indirect Immunoinfluscent Assay); NPLA (neutralizing peroxidase-linked antibody Assay), Molecular Biology: Quick Detection of Test Paper; PCR (Polymerase Chain Reaction); RT-PCR (Reverse Transcription PCR). Season*: Spring: Mar. to May; Summer: Jun. to Aug.; Autumn: Sep. to Nov.; Winter: Dec. to Feb. Age*: adult (> 6 month) and juvenile (≤ 6 month). Incorporate article in Supplementary Figures.

The prevalence of CDV in minks, foxes and raccoon dogs was 10.3% before 2000. The reason for the low prevalence may be related to the policies of some countries toward minks, foxes and raccoon dogs. For example, the UK amended the “European Convention for the Protection of Farm Animals “in 1992 (18). The United States Federal Government enacted the Animal Welfare Act in 1966, followed by the Improving Laboratory Animal Standards Act in 1985, and the EU introduced the animal welfare related bill “Council Directive 98 /58 /EC “in 1998 (19). However, there was no significant difference between the prevalence rates from 2000 to 2010 and that from 2010 to the present. There are also many countries that have introduced relevant welfare bills, but the popularity of CD is still spreading in various countries. Our analysis showed that as the improvement of economic level, people’s demand for fur animals is gradually increasing. In European social cognition, people attach great importance to fur, believing that it can not only keep warm and cover the body, but also show their status and wealth (20). People should not only focus on the economic value of fur-bearing animals, but also regulate breeding and prevent CD (Table 4).

**Table 4:** Estimation of prevalence rate of canine distemper small carnivores in various countries.

Countries	No. studies	Region	No. tested	No. positive	% Prevalence	% (95% CI)
China	12	Asia	4,937	1,278	25.9%	19.8–48.3
Japan	2	Asia	339	48	14.2%	9.4–29.2
Korea	2	Asia	196	129	65.8%	83.3–95.8
France	1	Europe	280	24	8.6%	5.9–12.6
Germany	4	Europe	1775	282	15.9%	2.0–28.1
Italy	1	Europe	133	51	38.3%	30.9–47.6
Luxembourg	1	Europe	61	8	13.1%	6.9–25.0
Norway	2	Europe	278	32	11.5%	8.3–16.0
Spanish	1	Europe	134	23	17.2%	11.8–24.9
USA	3	North America	198	46	23.2%	2.1–100
Argentina	2	South America	171	5	2.9%	1.3–7.1
Brazil	2	South America	80	11	13.8%	8.2–24.4
Total	33		8,582	1937	22.6%	11.4–24.8

This paper found that CDV is widely prevalent in minks, foxes and raccoon dogs, but the prevalence rate is the highest in raccoon dogs. This finding was similar to the prevalence previously found in German mink (21). The high prevalence of CDV may be related to the natural habitat of these species. Their proximity to human life makes them more likely to have direct or indirect contact with dogs infected with CDV (22). Studies have shown that in Germany, the CDV strains in dogs and free-range carnivores are the same, indicating that these dogs can be used as an external virus source for free-range populations (23). This was also the main reason for the spread of CDV in wild minks, foxes and raccoon dogs.

In the national group, South Korea had the highest positive rate. We think this may be related to the “breeding fever “of fur animals in South Korea in the 1920s (24). With regard to CDV in Italy, there have been several outbreaks of foxes (*Vulpes Vulpes*), badgers (*Meles Meles*) and minks in the alpine regions of northeastern Italy over the past decade, particularly in the Trentino-Al Adige, Veneto and Friuli-Venetian-Giulia region (25). The ease of transmission of the virus and its rapid arrival in the former alpine and urbanized areas of Italy, South Bavaria and Switzerland, which also leads to a high positive rate in Italy (26, 27). The United States, China as a big country of fur animal breeding with the increase of fur animal prices, breeding volume and breeding density are increasing (28). With the frequent introduction at home and abroad, this has caused great pressure on the prevention and control of CD (29). Although the CD vaccine has been widely used in China’s fur animal breeding industry, the mortality of fur animals such as minks, foxes and raccoon dogs caused by CDV remains high (29). According to the analysis of geographical grouping, we found that the prevalence of CDV was higher in the range of longitude 120E-180 and latitude 30-60 N. Through our study, we found that Asia and North America are just in this latitude and longitude range. At the same time, we believe that the two continents in the temperate monsoon climate have similar climatic characteristics. The annual temperature difference is large and the winter is cold. This is consistent with our findings - higher incidence in winter. But this is not consistent with the results of Dorji (30). The prevalence of CD was higher in winter. We combined the latitude and longitude, season and other factors to infer that this may be related to the breeding mode of minks, foxes and raccoon dogs. For farmers who raise fur animals for a living, the high incidence of CD can cause significant economic losses.

In the breeding mode subgroup, the positive rate of farms was the highest. CDV can be transmitted mainly through direct contact with diseased animals or through air or food (31). Mink, fox and raccoon dog currently account for a large proportion of economic animal farms, and large-scale cage breeding has become the mainstream breeding method of farms. We analyzed that the high breeding density of minks, foxes and raccoon dogs in the farm and the untimely ventilation led to the high prevalence of CD. In addition, some disinfectants have a killing effect on CDV (32). We suggest that farms should be kept clean, use disinfectant to clean the breeding room, reduce breeding density and timely ventilation play an important role in controlling CD.

A subpopulation analysis revealed that minks, foxes, and raccoon dogs with a population size of less than 500 were more susceptible to CDV. The wild minks, foxes and raccoon dogs that hunt small animals in small groups may break into human territory in order to hunt and contact with domestic dogs, which will cause the spread of CDV. Rural areas are often habitats for wild carnivores. These pathogens are often transmitted from domestic dogs to wild carnivores through occasional contact. These animals may be susceptible to CDV. In addition, wild carnivores are usually small in number and low in density (33). Therefore, they are often not suitable for maintaining the infection of highly pathogenic pluripotent viruses such as CDV (34). This finding is consistent with previous research, suggesting a potential contributing factor to the current epidemic of canine distemper. It is advisable for dog owners to maintain their pets’ vaccination schedules and minimize their contact with wildlife.

In age group, there was no significant difference in the prevalence minks, foxes and raccoon dogs between young (less than 6 months) and adult (more than 6 months). We speculate that due to the large number of minks, foxes and raccoon dogs; adult female and male animals are vaccinated, while young animals obtain natural antibodies through breast milk. Since the promotion and application of CD vaccine and mink parvovirus enteritis vaccine in the 1880s, CD and mink parvovirus enteritis in minks, foxes and raccoon dogs in the immune area have been better controlled (35). Therefore, we suggest that more attention should be paid to young animals, and timely vaccination should be given. It is of great significance to reduce the prevalence of CD by universal vaccination.

In the species subgroup, the prevalence of raccoon dogs was much higher than that of foxes and minks. Raccoon dogs were introduced from Russia to South Korea in the late 1920s for the production of fur. With the prosperity of silver fox and goat fur farms in Asian countries, the raccoon dog industry in South Korea has rapidly shrunk (32). Raccoon dogs living in fur farms escaped from Korea and became wild animals (36). Due to the absence of natural enemies, the population density of raccoon dogs has increased. If exposed to infected carnivores, it is possible to spread disease between domestic carnivores and wild raccoon dogs (37). This is one reason why the prevalence of CD in Korea is as high as 65.8% (95% CI, 83.3–95.8). We should control the scale of breeding and check the facilities regularly.

Most of the included studies used serological detection and molecular biological detection. The sensitivity and specificity of molecular biology are significantly higher than those of serology (38). Therefore, we analyze that the reason for the low positive rate of serological detection may be related to the frequent occurrence of false positive reactions during the detection process and the reduction of detection sensitivity (39). These methods are the mainstream CD diagnosis methods. We suggest that the detection method should be reasonably selected to reduce the occurrence of error and false positive reaction. In the collection of samples, we found that there was no significant difference in plasma, tissue and secretion, which may be related to the detection method. Our regression analysis shows that these methods have significant differences in reported prevalence and may be an important source of heterogeneity in this analysis.

We evaluated the global prevalence of CDV infection in minks, foxes and raccoon dogs through a meta-analysis of 33 systems. CD is very harmful to the fur industry. Sampling year, regional distribution, geographical factors, feeding patterns, detection methods and other factors affect the prevalence of CDV infection. It is suggested to reduce the contact of domestic dogs, carry out technical training and improve the technical level according to the feeding methods, geographical factors and climatic environment in different regions. In addition, comprehensive control strategies are adopted, such as disease prevention in various places. In addition, comprehensive control strategies such as disease prevention, immunization, quarantine and disinfection should also be standardized. We believe that due to widespread vaccination, the prevalence of the disease on farms is very low, so timely vaccination has a good control effect on the spread of CDV. In addition, in order to further explore the factors of mink, fox and raccoon dog infected with CD, it is necessary to carry out detailed epidemiological investigation in more areas.

There were three limitations to this study. First, when determining the search methodology, we attempted to create multiple databases to obtain more comprehensive articles, but there may have been research omissions due to database and language limitations. Second, the small number of studies from North and South America may affect the analysis of results in these regions. Third, the lack of some information (such as whether minks, foxes and raccoon dogs have fever and diarrhea) will affect the analysis results. However, we believe that this analysis can reflect the real epidemic situation of CD infection in minks, foxes and raccoon dogs on all continents.

## Data availability statement

The datasets presented in this study can be found in online repositories. The names of the repository/repositories and accession number(s) can be found in the article/Supplementary material.

## Author contributions

JL: Writing – review & editing, Writing – original draft, Visualization, Validation, Supervision, Software, Resources, Project administration, Methodology, Investigation, Funding acquisition, Formal analysis, Data curation, Conceptualization. TW: Writing – original draft, Validation, Supervision, Software, Project administration, Methodology, Investigation, Formal analysis, Data curation, Conceptualization. QW: Writing – review & editing, Writing – original draft, Visualization, Supervision, Software, Resources, Methodology, Investigation, Formal analysis, Data curation, Conceptualization. XW: Writing – review & editing, Writing – original draft, Supervision, Software, Methodology, Investigation, Formal analysis, Data curation, Conceptualization. XF: Writing – review & editing, Writing – original draft, Supervision, Software, Methodology, Investigation, Data curation, Conceptualization. TH: Writing – review & editing, Writing – original draft, Validation, Supervision, Software, Methodology, Investigation, Data curation, Conceptualization. XL: Writing – review & editing, Writing – original draft, Visualization, Validation, Supervision, Software, Resources, Project administration, Methodology, Investigation, Funding acquisition, Formal analysis, Data curation, Conceptualization. SK: Writing – review & editing, Writing – original draft, Supervision, Software, Methodology, Investigation, Data curation, Conceptualization. JL: Writing – review & editing, Writing – original draft, Supervision, Software, Methodology, Investigation, Data curation, Conceptualization. QG: Writing – review & editing, Writing – original draft, Visualization, Validation, Supervision, Software, Resources, Project administration, Methodology, Investigation, Data curation, Conceptualization. RD: Writing – review & editing, Writing – original draft, Visualization, Validation, Supervision, Software, Resources, Project administration, Methodology, Investigation, Funding acquisition, Formal analysis, Data curation, Conceptualization.
