# Supplementary material for: Prevalence of canine distemper in minks, foxes and raccoon dogs from 1983 to 2023 in Asia, North America, South America and Europe
Source: Front Vet Sci. 2024 Aug 13;11:1394631. doi: 10.3389/fvets.2024.1394631 (PMC11348944; doi:10.3389/fvets.2024.1394631)
Supplement: Supplementary file 1 [file Data_Sheet_1.pdf]

Table S1. PRISMA Checklist item.

Section/topic	#	Checklist item	Reported on page #
**TITLE**			
Title	1	Prevalence of Canine distemper in minks, foxes and raccoon dogs from 1983 – 2023 in Asia, North America, South America and Europe	
**ABSTRACT**			
Structured summary	2	Provide a structured summary including, as applicable: background; objectives; data sources; study eligibility criteria, participants, and interventions; study appraisal and synthesis methods; results; limitations; conclusions and implications of key findings; systematic review registration number.	
**INTRODUCTION**			
Rationale	3	Describe the rationale for the review in the context of what is already known.	
Objectives	4	Provide an explicit statement of questions being addressed with reference to participants, interventions, comparisons, outcomes, and study design (PICOS).	
**METHODS**			
Protocol and registration	5	Indicate if a review protocol exists, if and where it can be accessed (e.g., Web address), and, if available, provide registration information including registration number.	
Eligibility criteria	6	Specify study characteristics (e.g., PICOS, length of follow-up) and report characteristics (e.g., years considered, language, publication status) used as criteria for eligibility, giving rationale.	
Information sources	7	Describe all information sources (e.g., databases with dates of coverage, contact with study authors to identify additional studies) in the search and date last searched.	
Search	8	Present full electronic search strategy for at least one database, including any limits used, such that it could be repeated.	

Study selection	9	State the process for selecting studies (i.e., screening, eligibility, included in systematic review, and, if applicable, included in the meta-analysis).	
Data collection process	10	Describe method of data extraction from reports (e.g., piloted forms, independently, in duplicate) and any processes for obtaining and confirming data from investigators.	
Data items	11	List and define all variables for which data were sought (e.g., PICOS, funding sources) and any assumptions and simplifications made.	
Risk of bias in individual studies	12	Describe methods used for assessing risk of bias of individual studies (including specification of whether this was done at the study or outcome level), and how this information is to be used in any data synthesis.	
Summary measures	13	State the principal summary measures (e.g., risk ratio, difference in means).	
Synthesis of results	14	Describe the methods of handling data and combining results of studies, if done, including measures of consistency (e.g., I^2) for each meta-analysis.	
Risk of bias across studies	15	Specify any assessment of risk of bias that may affect the cumulative evidence (e.g., publication bias, selective reporting within studies).	
Additional analyses	16	Describe methods of additional analyses (e.g., sensitivity or subgroup analyses, meta-regression), if done, indicating which were pre-specified.	
**RESULTS**			
Study selection	17	Give numbers of studies screened, assessed for eligibility, and included in the review, with reasons for exclusions at each stage, ideally with a flow diagram.	Table 1, Figure 1 and 2
Study characteristics	18	For each study, present characteristics for which data were extracted (e.g., study size, PICOS, follow-up period) and provide the citations.	Table 2, Figure 2
Risk of bias within studies	19	Present data on risk of bias of each study and, if available, any outcome level assessment (see item 12).	Figure 3, Figure 4, Figure 5, Figure 6and Figure S3-9
Results of individual studies	20	For all outcomes considered (benefits or harms), present, for each study: (a) simple summary data for each intervention group (b) effect estimates and confidence intervals, ideally with a forest plot.	Table 3, Figure 7

Synthesis of results	21	Present results of each meta-analysis done, including confidence intervals and measures of consistency.	Figure 2
Risk of bias across studies	22	Present results of any assessment of risk of bias across studies (see Item 15).	Table 3
Additional analysis	23	Give results of additional analyses, if done (e.g., sensitivity or subgroup analyses, meta-regression [see Item 16]).	Table 3
**DISCUSSION**			
Summary of evidence	24	Summarize the main findings including the strength of evidence for each main outcome; consider their relevance to key groups (e.g., healthcare providers, users, and policy makers).	
Limitations	25	Discuss limitations at study and outcome level (e.g., risk of bias), and at review-level (e.g., incomplete retrieval of identified research, reporting bias).	
Conclusions	26	Provide a general interpretation of the results in the context of other evidence, and implications for future research.	
**FUNDING**			
Funding	27	Describe sources of funding for the systematic review and other support (e.g., supply of data); role of founders for the systematic review.	

From: Moher D, Liberati A, Tetzlaff J, Altman DG, The PRISMA Group (2009). Preferred Reporting Items for Systematic Reviews and Meta-Analyses: The PRISMA Statement. PLoS Med 6(6): e1000097. doi:10.1371/journal.pmed1000097

For more information, visit: **www.prisma-statement.org**.

Table S2. The code in R for meta-analysis.

No transformation<br>(PRAW)	rate<-transform[m1, r= event/n];<br>shapiro.test(rate$r)
Logarithmic conversion<br>(PLN)	rate<-transform [m1, log=log(event/n)];<br>shapiro.test(rate$log)
Logit transformation<br>(PLOGIT)	rate<-transform{m1, logit=log[(event/n)/(1-event/n)]};<br>shapiro.test(rate$logit)
Arcsine transformation<br>(PAS)	rate<-transform{m1, arcsin.size=asin[sqrt(event/(n+1))]};<br>shapiro.test(rate$arcsin)
Double-arcsine<br>transformation (PFT)	rate<-<br>transform{m1,darcsin=0.5*[asin(sqrt(event/(n+1)))+asin((sqrt(event+1)/<br>(n+1)))]};<br>shapiro.test(rate$darcsin)
Forest plots	forest [meta1, xlim=c(-0.2, 1)]
Funnel chart	funnel (meta1)

Egger's test	metabias (meta1, method="linreg")
The sensitivity analysis	metainf (meta1, pooled = "random") forest (metainf (meta1, pooled = "random"), xlim=c(0, 0.4))
Subgroup analysis	meta1<-metaprop(event, n, study, data=rate, sm="PLN", incr=0.5, allincr=TRUE, addincr=FALSE, title="", byvar= subgroup title, print.byvar=TRUE)
Meta-regression analysis	metareg (meta1, ~covariate title)

Table S3. Included studies and quality scores**.**

	**Reference ID**	**No. tested**	**No. positive**	**Prevalence**	**Random sampling or not**	**Sampled method detailly or not**	**Sampled method detailly or not**	**Sample time clearly or not**	**Four or more risk factors or not**	**Score**
1	Feng et al. (2011)	847	324	0.382526564	N	Y	N	Y	Y	3
2	Dai et al. (2014)	100	34	0.34	N	y	Y	Y	Y	4
3	Dai (2015)	100	35	0.35	N	Y	Y	Y	Y	4
4	Liu (2009)	49	22	0.448979592	N	Y	N	Y	Y	3
5	Luo et al. (2008)	380	88	0.231578947	N	Y	N	Y	Y	3
6	Wang et al. (2013)	280	112	0.4	N	Y	N	Y	Y	3

7	Wang et al. (2013)	996	223	0.223895582	N	N	Y	Y	Y	3
8	Pang (2018)	379	44	0.116094987	N	Y	N	N	Y	2
9	Zong (2015)	566	96	0.169611307	N	Y	N	Y	Y	3
10	Zhang et al. (2016)	1065	165	0.154929577	N	Y	N	Y	Y	3
11	Zhu (2020)	90	54	0.6	N	Y	N	N	Y	2
12	Akerstedt J et al. (2010)	100	12	0.12	N	Y	N	N	Y	2
13	Amundson TE et al. (1981)	57	6	0.105263158	N	Y	Y	Y	Y	4
14	Zhang et al. (2015)	85	81	0.952941176	Y	Y	N	N	Y	3
15	Cha SY et al. (2012)	102	45	0.441176471	N	Y	N	Y	Y	3
16	Damien BC et al. (2002)	61	8	0.131147541	N	Y	Y	Y	Y	4
17	Denzin N et al.(2013)	761	232	0.304862024	N	Y	N	Y	Y	3
18	Frölich K et al. (2000)	601	32	0.053244592	N	Y	N	Y	Y	3
19	Furtado MM et al. (2016)	58	7	0.120689655	N	Y	Y	Y	Y	4
20	Kameo Y et al. (2012)	20	5	0.25	N	Y	N	Y	Y	3
21	Martino PE et al. (2017)	87	2	0.022988506	N	Y	N	Y	Y	3
22	Yang DK et al. (2013)	94	84	0.893617021	N	Y	N	Y	Y	3
23	Lempp C et al. (2017)	30	1	0.033333333	Y	Y	N	Y	Y	4
24	McCue PM and O'Farrell TP (1988)	100	4	0.04	N	Y	Y	Y	Y	4
25	Martino PE et al. (2004)	84	3	0.035714286	N	Y	Y	Y	Y	4
26	Weber MN et al. (2020)	22	4	0.181818182	N	Y	N	Y	Y	3
27	Tryland M et al. (2018)	178	20	0.112359551	N	Y	N	Y	Y	3
28	Philippa J et al. (2008)	280	24	0.085714286	N	Y	Y	Y	Y	4
29	Sobrino R et al. (2008)	134	23	0.171641791	N	Y	N	Y	Y	3
30	Suzuki J et al. (2015)	319	43	0.134796238	N	Y	N	Y	Y	3
31	Timm SF et al. (2009)	41	36	0.87804878	N	Y	N	Y	Y	3
32	Truyen U et al. (1998)	383	17	0.044386423	N	Y	N	Y	Y	3
33	Trogu T et al. (2021)	133	51	0.383458647	N	Y	N	Y	Y	3

Y*: Yes; N*: No.

S4 Table. Egger's for publication bias

slope	bias	se. bias	t	df	P-value
**-0.1283089**	**-9.0754610**	**1.7399418**	**-5.216**	**31**	**1.156e-05**

References

Akerstedt J., Lillehaug A., Larsen I.L., Eide N.E., Arnemo J.M., Handeland K., 2010. Serosurvey for canine distemper virus, canine adenovirus, Leptospira interrogans, and Toxoplasma gondii in free-ranging canids in Scandinavia and Svalbard. J. Wildl. Dis. 46, 474-80. https://doi.org/10.7589/0090-3558-46.2.474.

Amundson T.E., Yuill T.M., 1981. Prevalence of selected pathogenic microbial agents in the red fox (Vulpes fulva) and gray fox (Urocyon cinereoargenteus) of southwestern Wisconsin. J. Wildl. Dis. 17, 17-22. https://doi.org/10.7589/0090-3558-17.1.17

Cha S.Y., Kim E.J., Kang M., Jang S.H., Lee H.B., Jang H.K., 2012. Epidemiology of canine distemper virus in wild raccoon dogs (Nyctereutes procyonoides) from South Korea. Comp. Immunol. Microbiol. Infect. Dis. 35, 497-504. https://doi.org/10.1016/j.cimid.2012.04.006.

Dai X.M., 2015. Prevalence of canine distemper in fur-bearing animals and biological characterization of a strain of canine distemper virus. Shandong. Agricultural. University. (In Chinese)

Dai X.M., Wang Q.M., Hui R.Q., Wang Q.J., Chang W.S., 2014. Detection of canine distemper antibody in mink and its analysis. Shandong. J. Anim. Sci. Vet. Med. 04, 10-1. (In Chinese)

Damien B.C., Martina B.E., Losch S., Mossong J., Osterhaus A.D., Muller C.P., 2002. Prevalence of antibodies against canine distemper virus among red foxes in Luxembourg. J. Wildl. Dis. 38, 856-9. https://doi.org/10.7589/0090-3558-38.4.856.

Denzin N., Herwig V., van der Grinten E., 2013. Occurrence and geographical distribution of Canine Distemper Virus infection in red foxes (Vulpes vulpes) of Saxony-Anhalt, Germany. Vet. Microbiol. 162, 214-8. https://doi.org/10.1016/j.vetmic.2012.08.031.

Feng W.Y., Zhou Q.M., Huang J., Li G.W., Qin B., 2011. Study on the incidence pattern of canine distemper in blue foxes on large-scale farms. Contemp. Anim. Livest. 05, 27-8. (In Chinese)

Frölich K., Czupalla O., Haas L., Hentschke J., Dedek J., Fickel J., 2000. Epizootiological investigations of canine distemper virus in free-ranging carnivores from Germany. Vet. Microbiol. 74, 283-92. https://doi.org/10.1016/s0378-1135(00)00192-9.

Furtado M.M., Hayashi E.M., Allendorf S.D., Coelho C.J., de Almeida Jácomo A.T., Megid J., Ramos Filho J.D., Silveira L., Tôrres N.M., Ferreira Neto J.S., 2016. Exposure of Free-Ranging Wild Carnivores and Domestic Dogs to Canine Distemper Virus and Parvovirus in the Cerrado of Central Brazil. Ecohealth. 13, 549-57. https://doi.org/10.1007/s10393-016-1146-4.

Kameo Y., Nagao Y., Nishio Y., Shimoda H., Nakano H., Suzuki K., Une Y., Sato H., Shimojima M., Maeda K., 2011. Epizootic canine distemper virus infection among wild mammals. Vet. Microbiol. 2012 Jan 27;154(3-4):222-9. https://doi.org/10.1016/j.vetmic.2011.07.006.

Lempp C., Jungwirth N., Grilo M.L., Reckendorf A., Ulrich A., van Neer A., Bodewes R., Pfankuche V.M., Bauer C., Osterhaus A.D., Baumgärtner W., Siebert U., Pathological findings in the red fox (Vulpes vulpes), stone marten (Martes foina) and raccoon dog (Nyctereutes procyonoides), with special emphasis on infectious and zoonotic agents in Northern Germany. PLoS. One. 12, e0175469. https://doi.org/10.1371/journal.pone.0175469.

Liu T.T., 2018. Prevalence survey of major diseases in furbearer host communities during 2009-2017. Shandong. Agricultural. University. (In Chinese)

Luo G.L., Zhao J.J., Zhang H.L., Wang F.X., Cai X.L., Wu W., Yan X.J., Shao X.Q., Yi L., 2008. Survey and analysis of the occurrence and prevalence of major diseases of mink, fox and raccoon in China, 2006-2007. Spec. Wild. Econ. Anim. Plant. Research. 47, 71-4. (In Chinese)

Martino P.E., Montenegro J.L., Preziosi J.A., Venturini C., Bacigalupe D., Stanchi N.O., Bautista E.L., 2004. Serological survey of selected pathogens of free-ranging foxes in southern Argentina, 1998--2001. Rev. Sci. Tech. 23, 801-6. https://doi.org/10.20506/rst.23.3.1521.

Martino P.E., Samartino L.E., Stanchi N.O., Radman N.E., Parrado E.J., 2017. Serology and protein electrophoresis for evidence of exposure to 12 mink pathogens in free-ranging American mink (Neovison vison) in Argentina. Vet. Q. 37, 207-11. https://doi.org/10.1080/01652176.2017.1336810.

McCue P.M., O'Farrell T.P., 1988. Serological survey for selected diseases in the endangered San Joaquin kit fox (Vulpes macrotis mutica). J Wildl Dis. 24, 274-81. https://doi.org/10.7589/0090-3558-24.2.274.

Pang J.Y., 2018. Detection of ADV, MEV and CDV in mink carcasses in Shandong Province with variation of isolates and analysis of ADV safety in mice. Shandong. Agricultural. University. (In Chinese)

Philippa J., Fournier-Chambrillon C., Fournier P., Schaftenaar W., van de Bildt M., van Herweijnen R., Kuiken T., Liabeuf M., Ditcharry S., Joubert L., Bégnier M., Osterhaus A., 2008. Serologic survey for selected viral pathogens in free-ranging endangered European mink (Mustela lutreola) and other mustelids from south-western France. J. Wildl. Dis. 44, 791-801. https://doi.org/10.7589/0090-3558-44.4.791.

Sobrino R., Arnal M.C., Luco D.F., Gortázar C., 2007. Prevalence of antibodies against canine distemper virus and canine parvovirus among foxes and wolves from Spain. Vet. Microbiol. 126, 251-6. https://doi.org/10.1016/j.vetmic.2007.06.014.

Suzuki J., Nishio Y., Kameo Y., Terada Y., Kuwata R., Shimoda H., Suzuki K., Maeda K., 2015. Canine distemper virus infection among wildlife before and after the epidemic. J. Vet. Med. Sci. 77, 1457-63. https://doi.org/10.1292/jvms.15-0237.

Timm S.F., Munson L., Summers B.A., Terio K.A., Dubovi E.J., Rupprecht C.E., Kapil S., Garcelon D.K., 2009. A suspected canine distemper epidemic as the cause of a catastrophic decline in Santa Catalina Island foxes (Urocyon littoralis catalinae). J. Wildl. Dis. 45, 333-43. https://doi.org/10.7589/0090-3558-45.2.333.

Trogu T., Canziani S., Salvato S., Bianchi A., Bertoletti I., Gibelli L.R., Alborali G.L., Barbieri I., Gaffuri A., Sala G., Sozzi E., Lelli D., Lavazza A., Moreno A., 2021. Canine Distemper Outbreaks in Wild Carnivores in Northern Italy. Viruses. 13, 99. https://doi.org/10.3390/v13010099.

Truyen U., Müller T., Heidrich R., Tackmann K., Carmichael L.E., 1998. Survey on viral pathogens in wild red foxes (Vulpes vulpes) in Germany with emphasis on parvoviruses and analysis of a DNA sequence from a red fox parvovirus. Epidemiol. Infect. 121, 433-40. https://doi.org/10.1017/s0950268898001319.

Tryland M. , Balboni A., Killengreen S.T., Mørk T., Nielsen O., Yoccoz N.G., Ims R.A., Fuglei E., 2018. A screening for canine distemper virus, canine adenovirus and carnivore protoparvoviruses in Arctic foxes (Vulpes lagopus) and red foxes (Vulpes vulpes) from Arctic and sub-Arctic regions of Norway. Polar. Research. 37,

1498678. https://doi.org/10.1080/17518369.2018.1498678.

Wang A., Qin X.B., Shan H., 2013. Epidemiologic survey of mink diseases in Shandong Province, China. Anim. Livest. Vet. Med. 64, 86-8. (In Chinese)

Wang C., Bing Q.Z., Zhang Y., Huang J., Shan H., 2013. Epidemiologic investigation of canine distemper in mink. China. Anim. Livest. Vet. Med. 40, 198-201. (In Chinese)

Weber M.N., Mosena A.C.S., da Silva M.S., Canova R., de Lorenzo C., Olegário J.C., Budaszewski R.F., Baumbach L.F., Soares J.F., Sonne L., Varela A.P.M., Mayer F.Q., de Oliveira L.G.S., Canal C.W., 2020. Virome of crab-eating (Cerdocyon thous) and pampas foxes (Lycalopex gymnocercus) from southern Brazil and Uruguay. Infect. Genet. Evol. 85, 104421. https://doi.org/10.1016/j.meegid.2020.104421.

Yang D.K., Kim H.H., Nah J.J., Choi S.S., Kim J.T., Jeong W.H., Song J.Y., 2013. Serologic Survey of Rabies Virus, Canine Distemper Virus and Parvovirus in Wild Raccoon Dogs (Nyctereutes procyonoides koreensis) in Korea. J. Bacteriol. Virology. 43, 204-9. https://doi.org/10.4167/jbv.2013.43.3.204.

Zhang Y.H., Zhang Y.M., Zhu Q.Y., 2016. Epidemiologic investigation of canine distemper in mink. Spec. Econ. Anim. Plant. 19, 18-9. (In Chinese)

Zhang Y.Y., Lu H.P., Wang X.S., Guo Y.L., Wu N., Li Y.Y., Shi Q.M., 2015. ELISA for the detection of canine distemper antibodies in foxes. Agr. Sci. Technol. 16, 120-4. https://doi.org/10.16175/j.cnki.1009-4229.2015.01.032.

Zhu X.Y., Cai X.H., Lu R.G., Bu Y., Bai L., Zhu Y.Z., Lian S.Z., Bai X., Yan X.J., Hu B., 2021. Preliminary establishment of a semi-quantitative indirect ELISA method for the detection of IgG antibodies to mink mosaic virus. Chin. J. Vet. Sci. 41, 661-9. (In Chinese)

Zong X.C., 2015. Molecular epidemiological investigation of five viral diseases in mink in Shandong Province, China. Shandong. Agricultural. University. (In Chinese)

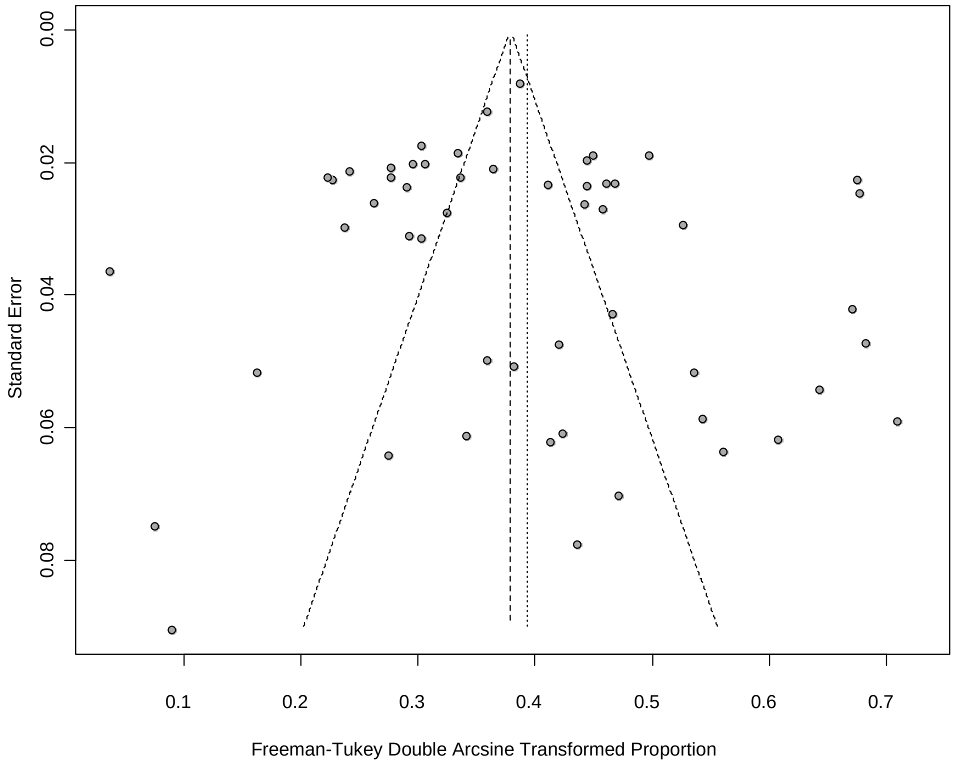

Figure S1. Funnel plot with pseudo 95% confidence limits intervals for the examination of publication bias of region

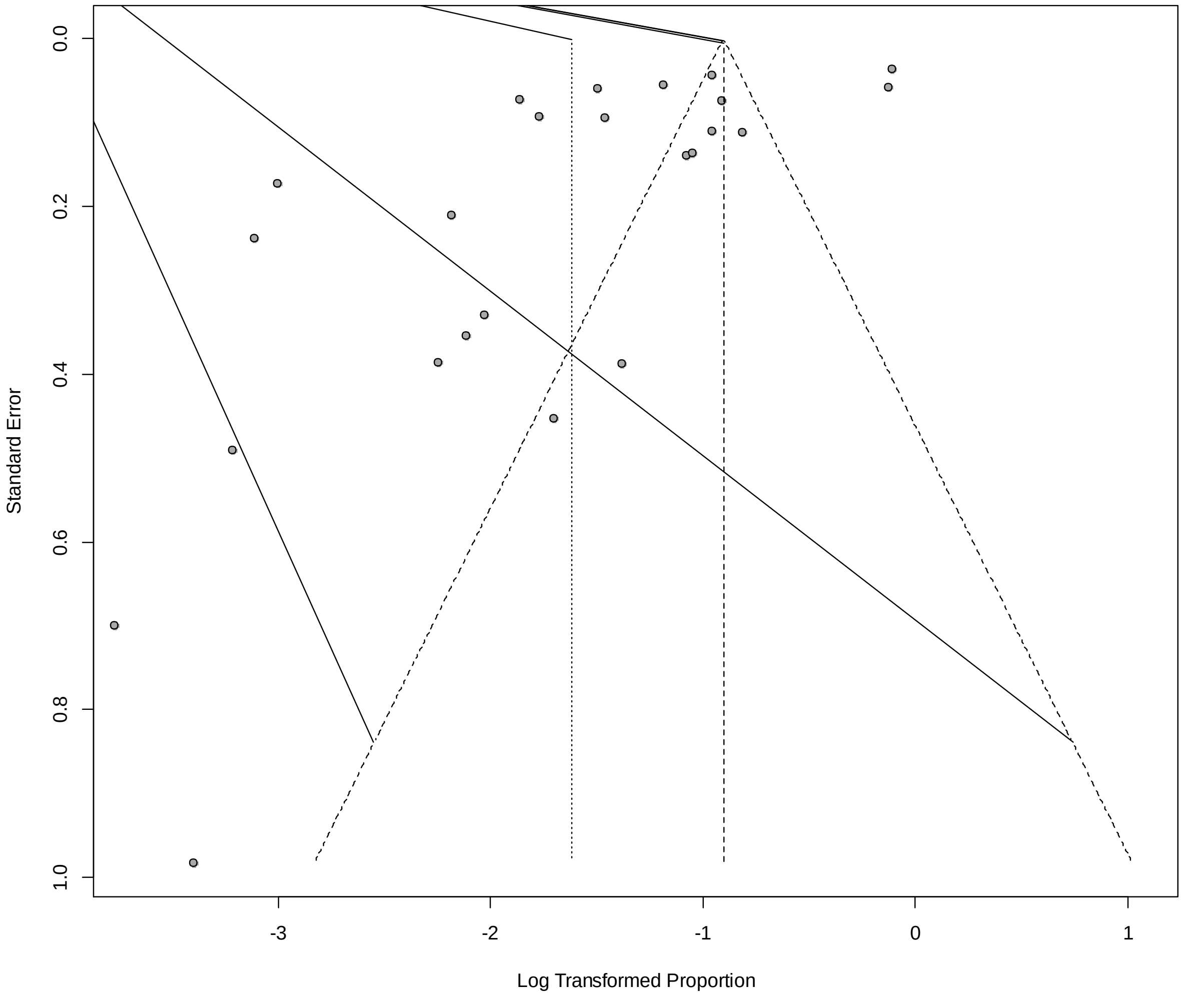

Figure S2. Funnel plot with pseudo 95% confidence limits intervals for the examination of publication bias of sampling years

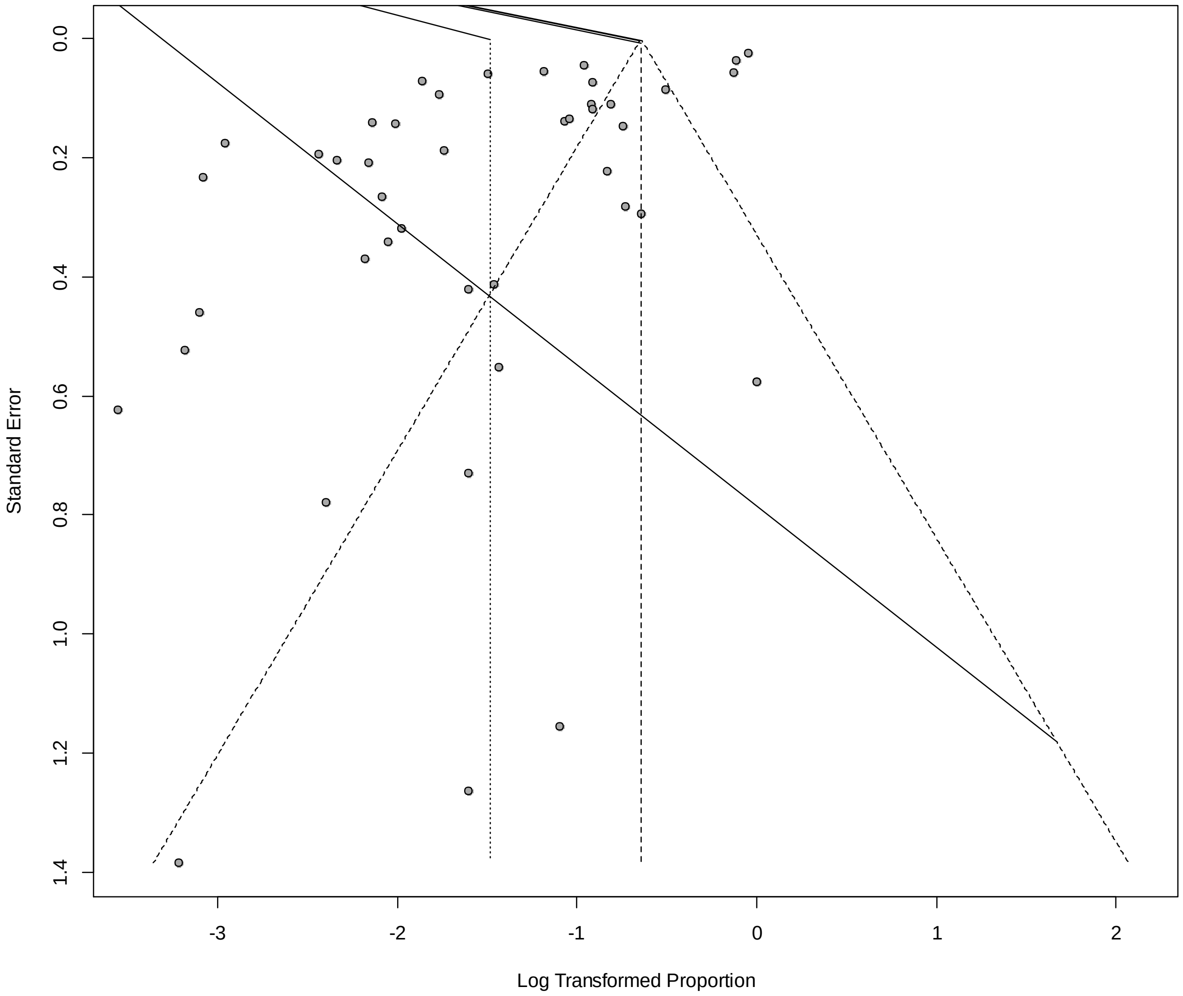

Figure S3. Funnel plot with pseudo 95% confidence limits intervals for the examination of publication bias of species

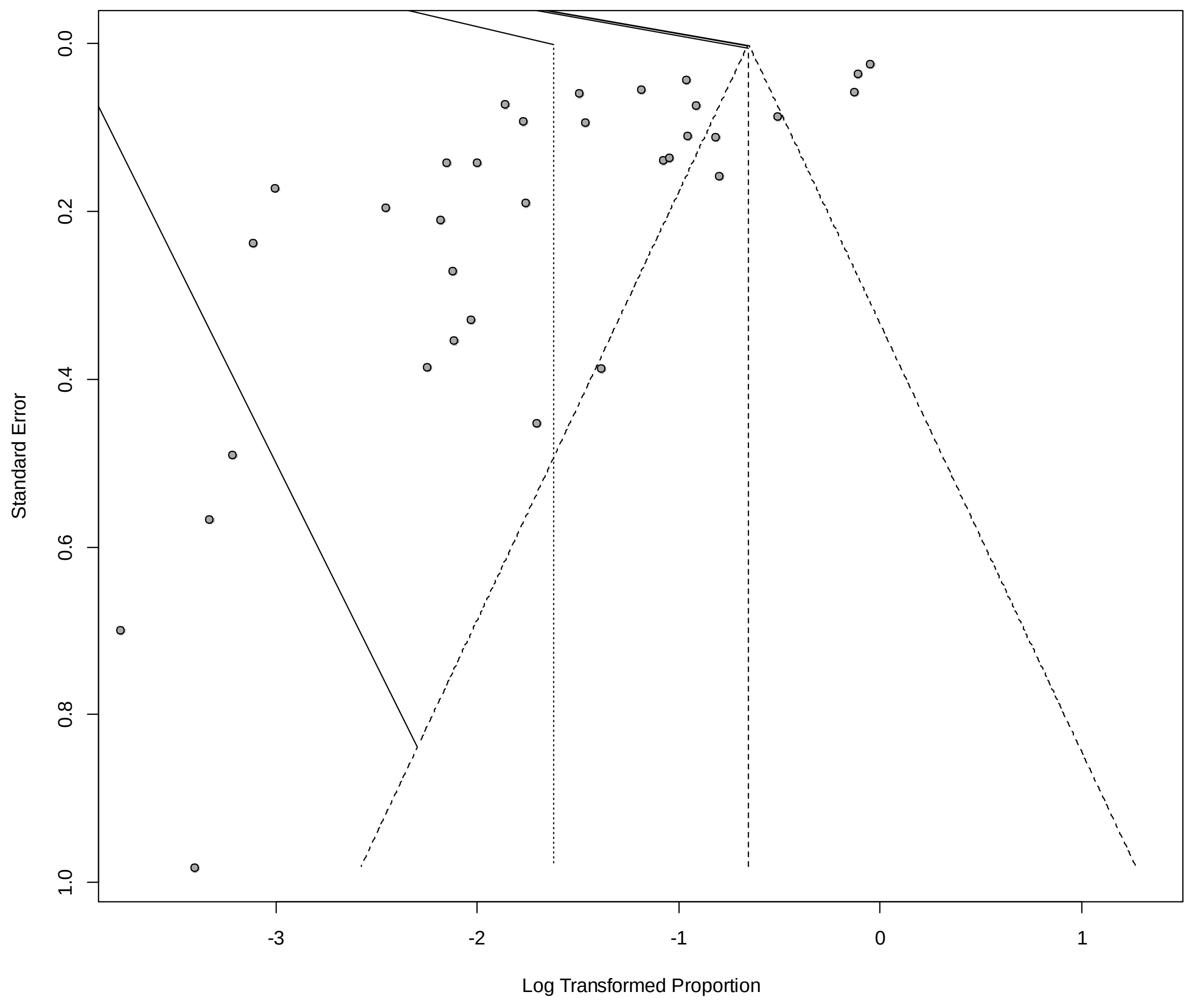

Figure S4. Funnel plot with pseudo 95% confidence limits intervals for the examination of publication bias of detection method

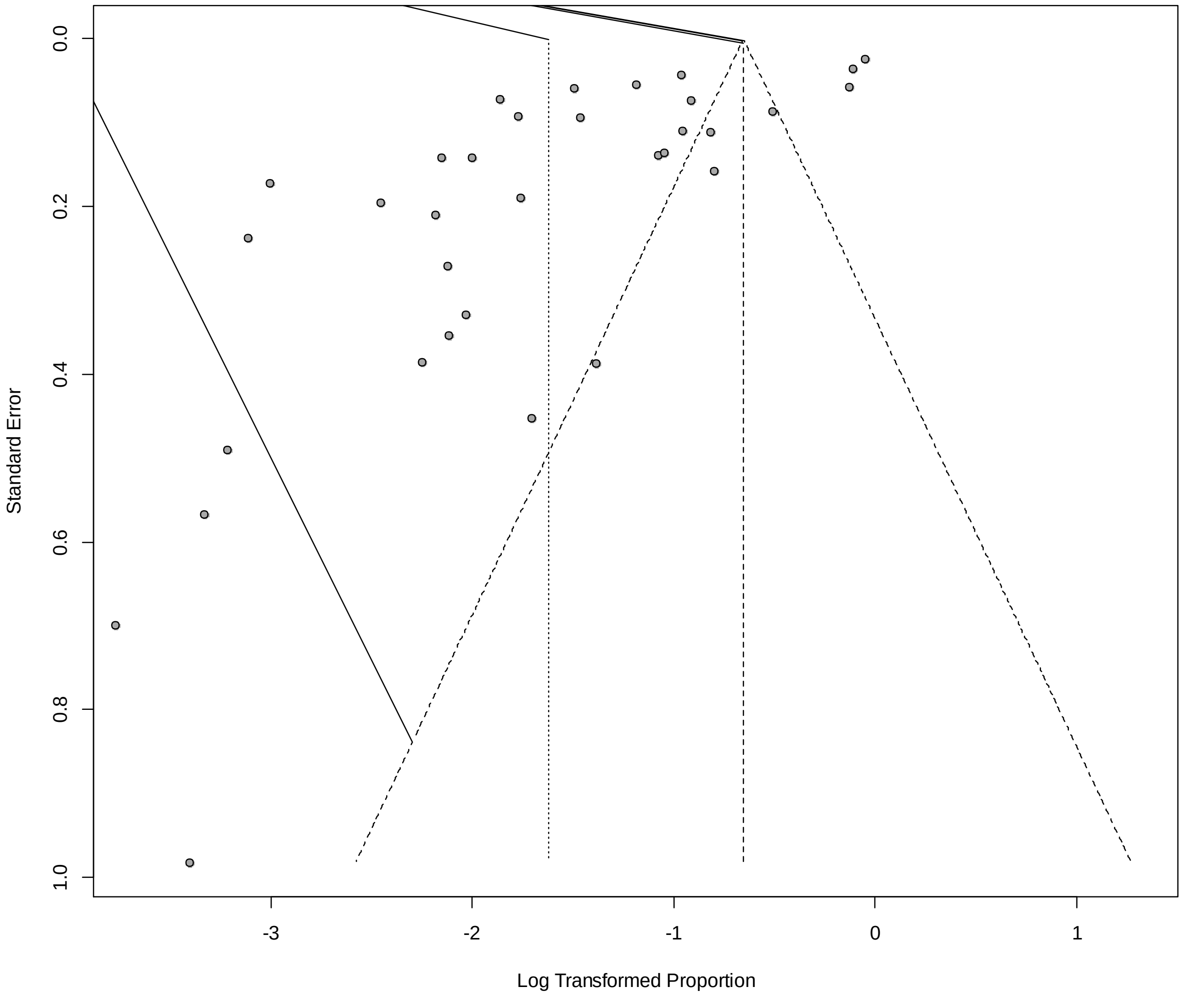

Figure S5. Funnel plot with pseudo 95% confidence limits intervals for the examination of publication bias of sample type

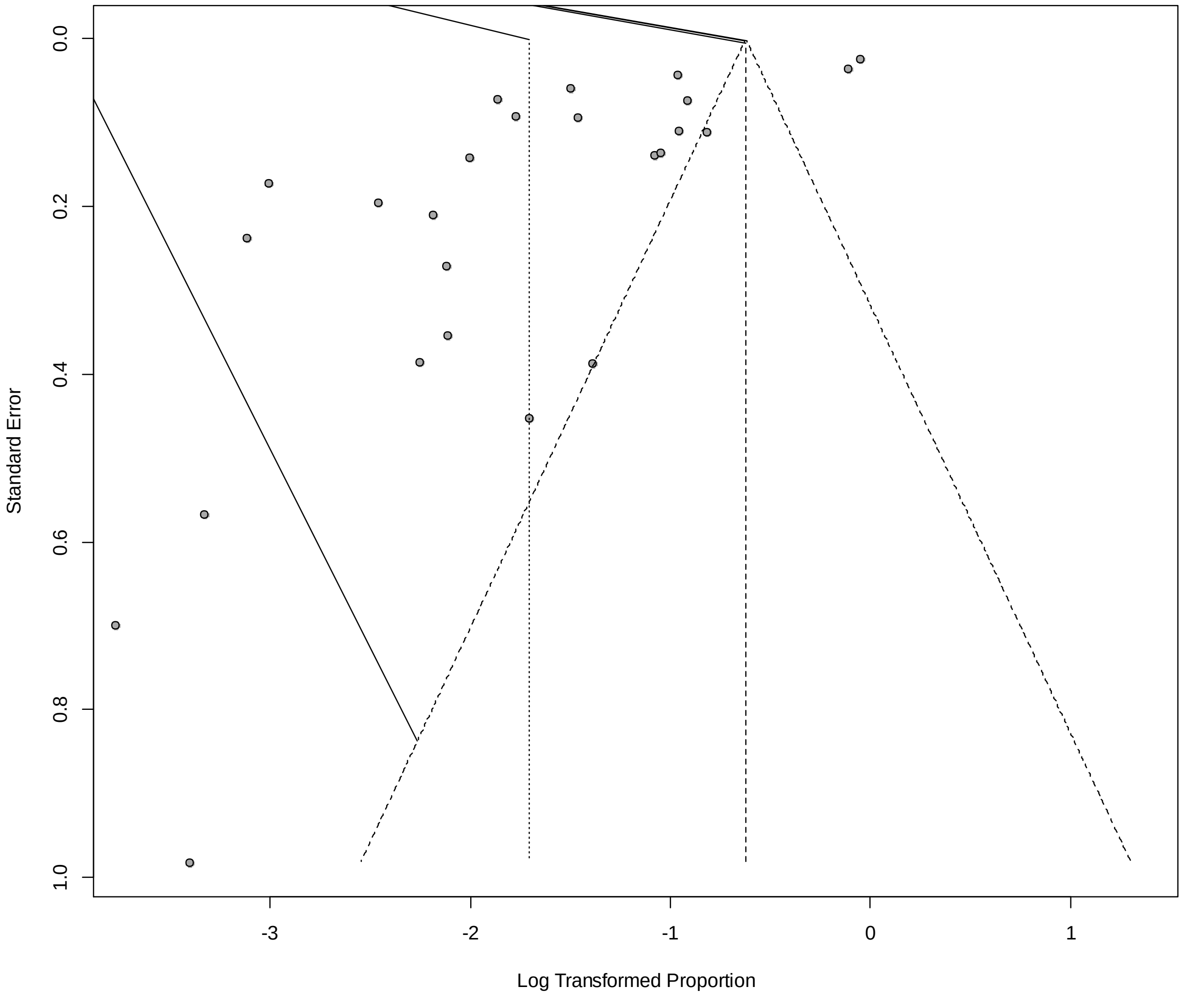

Figure S6. Funnel plot with pseudo 95% confidence limits intervals for the examination of publication bias of aquaculture model

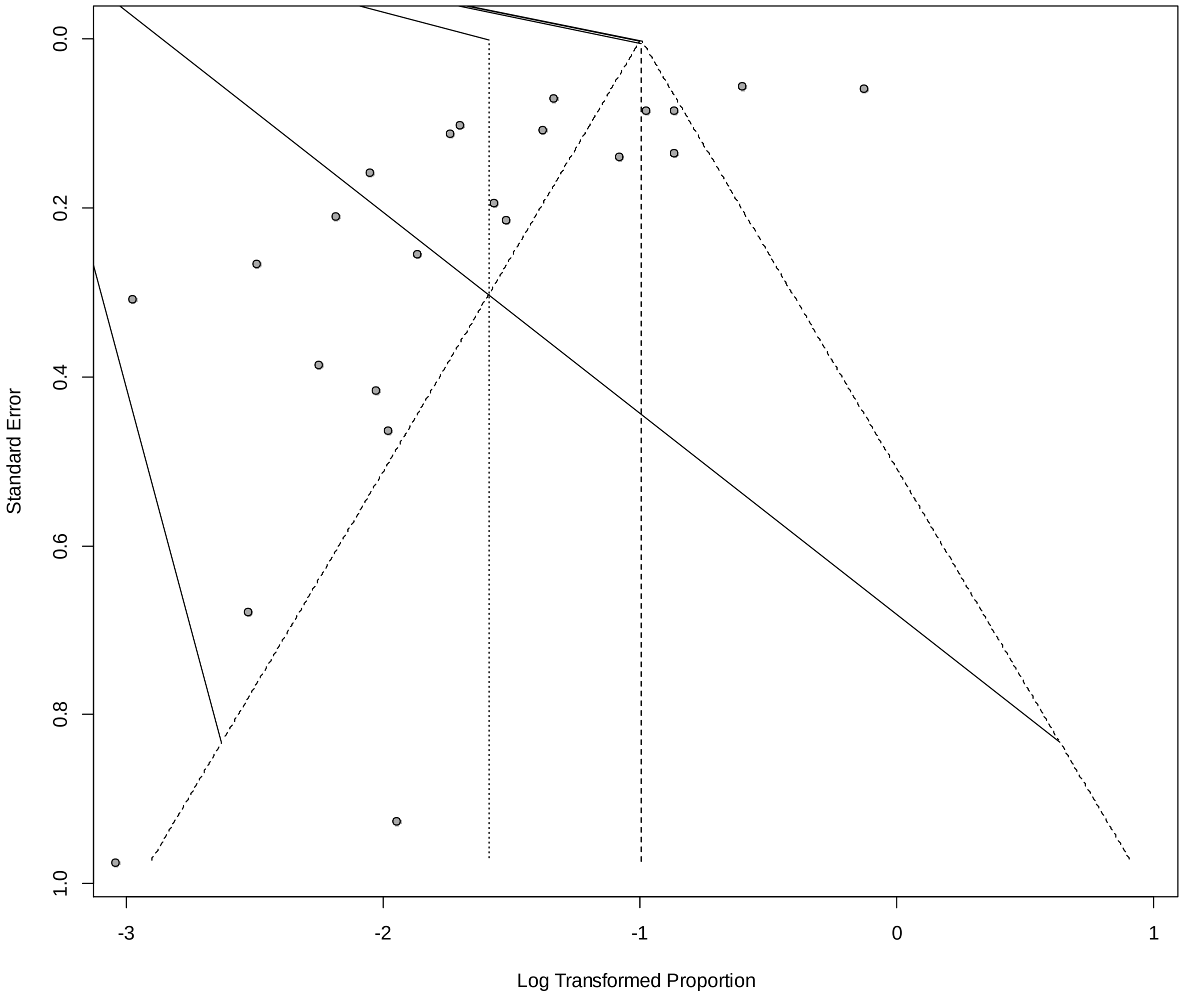

Figure S7. Funnel plot with pseudo 95% confidence limits intervals for the examination of publication bias of season

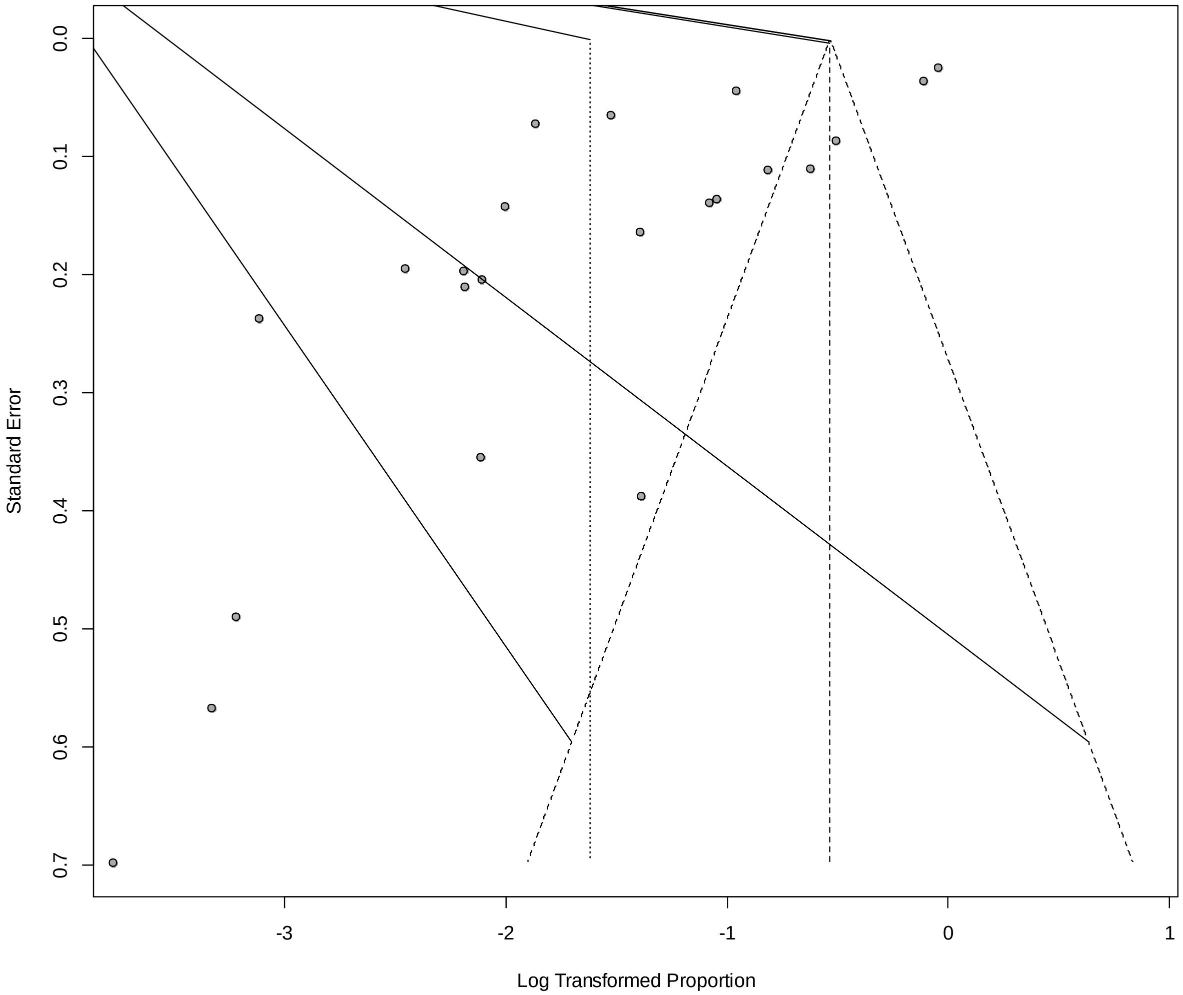

Figure S8. Funnel plot with pseudo 95% confidence limits intervals for the examination of publication bias of longitude

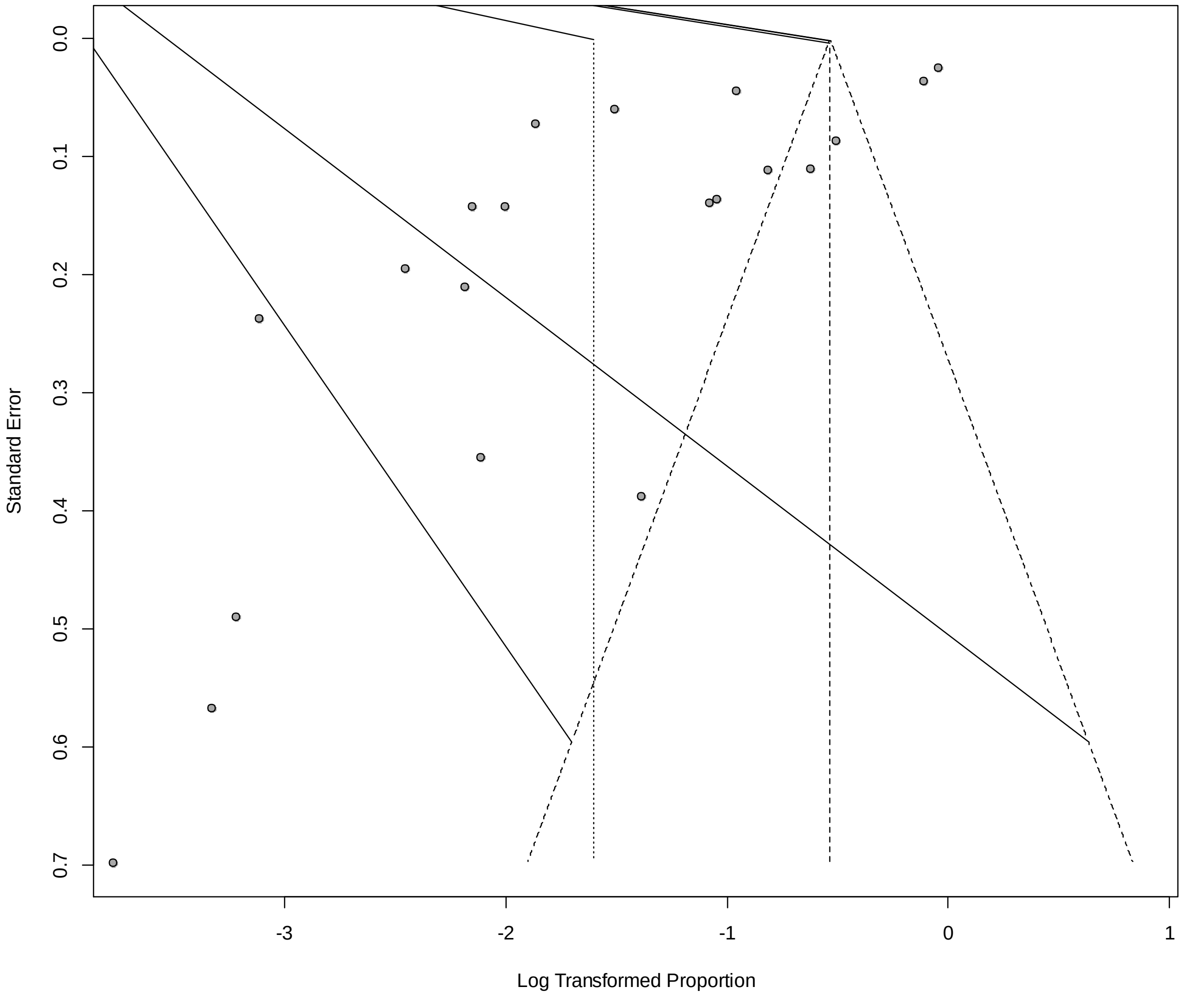

Figure S9. Funnel plot with pseudo 95% confidence limits intervals for the examination of publication bias of latitude

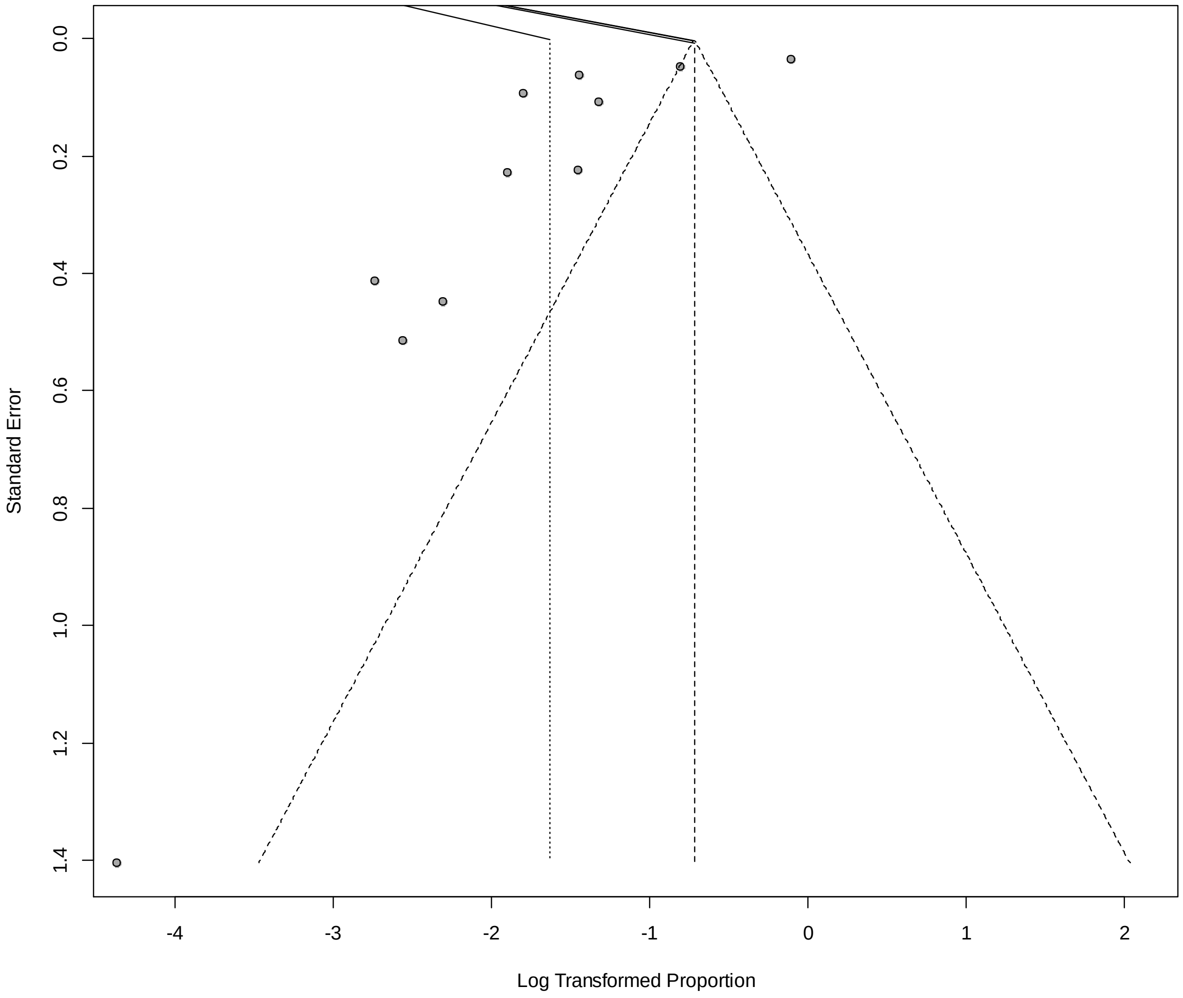

Figure S10. Funnel plot with pseudo 95% confidence limits intervals for the examination of publication bias of age

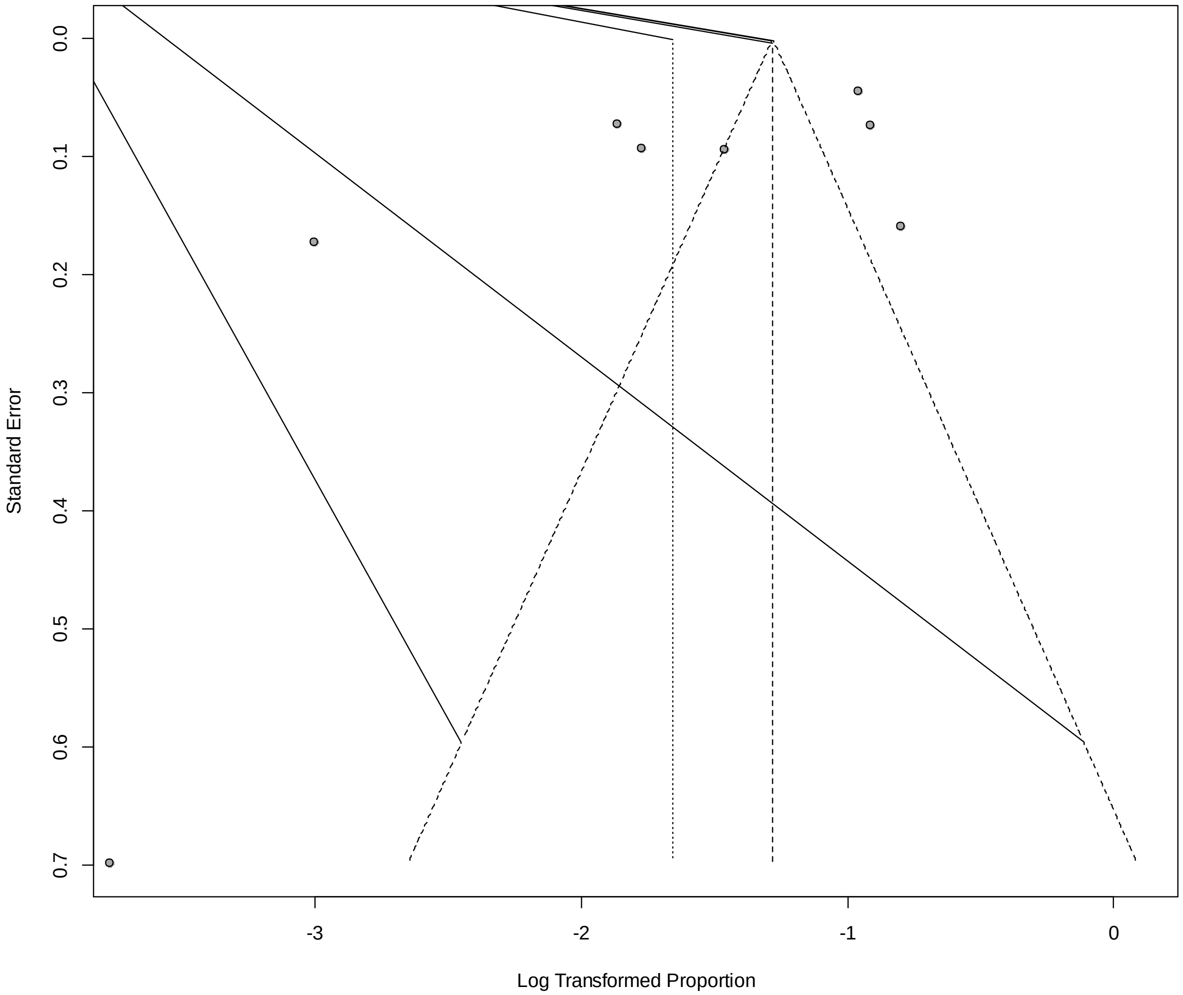

Figure S11. Funnel plot with pseudo 95% confidence limits intervals for the examination of publication bias of healthy condition

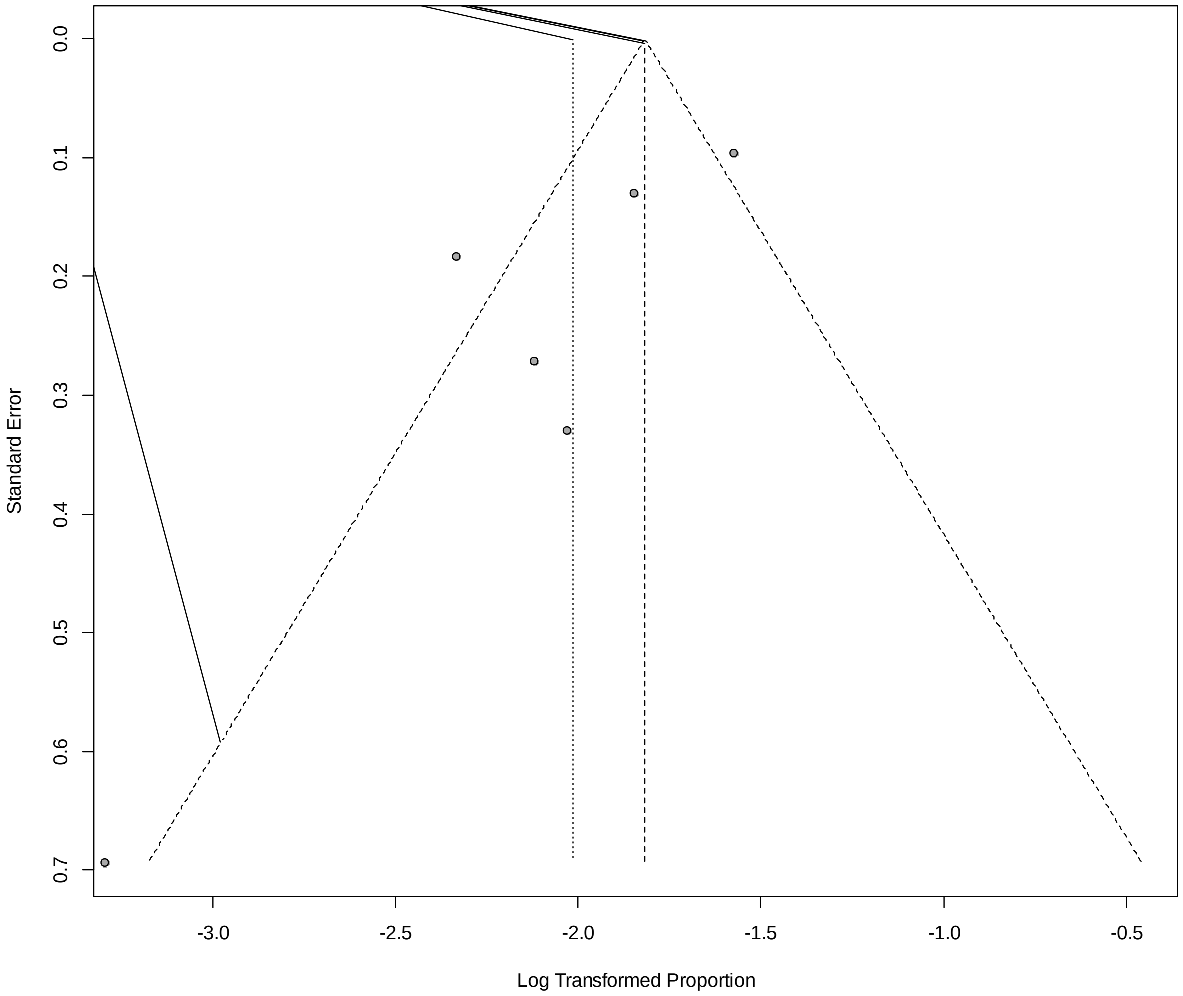

Figure S12. Funnel plot with pseudo 95% confidence limits intervals for the examination of publication bias of population size

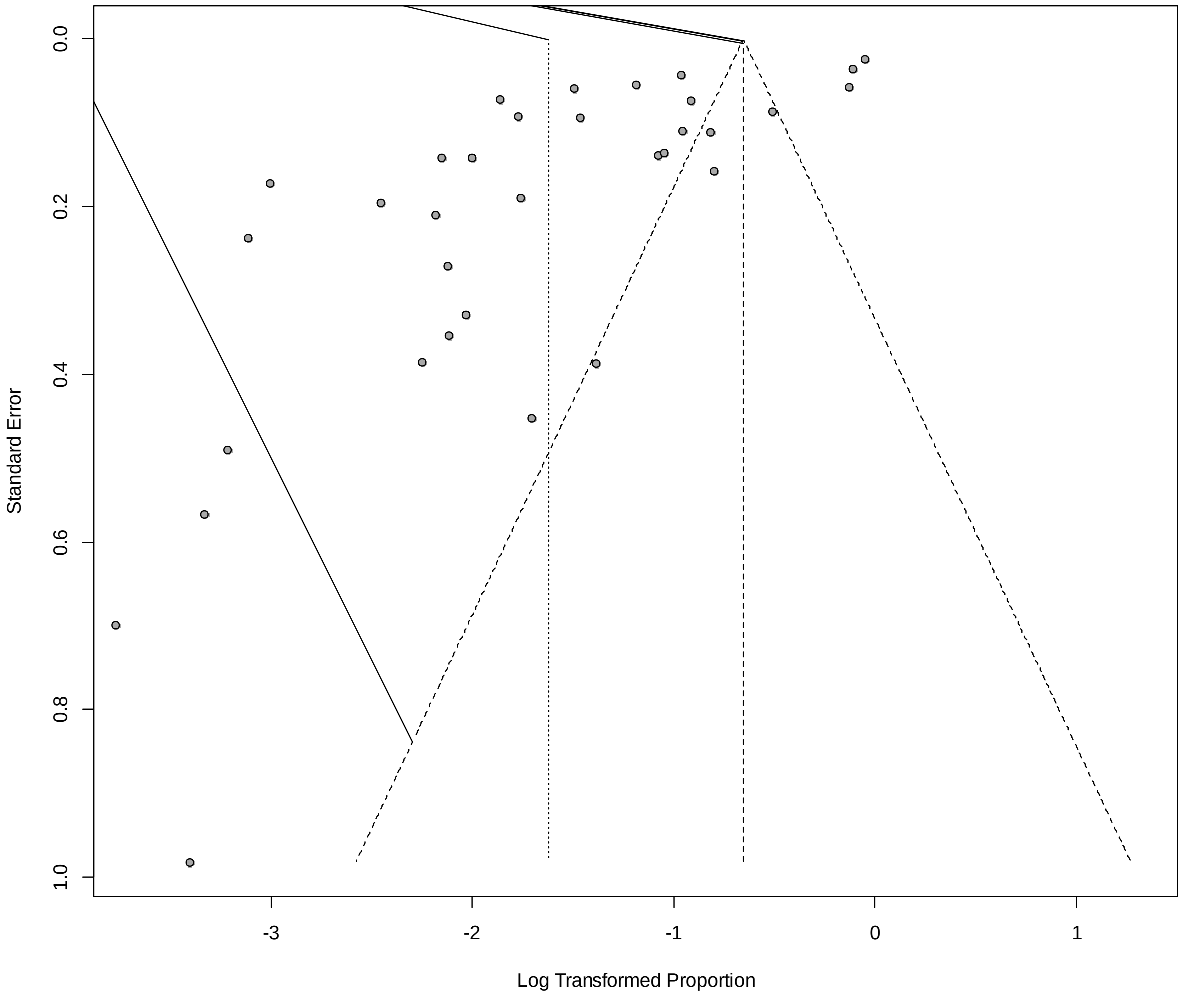

Figure S13. Funnel plot with pseudo 95% confidence limits intervals for the examination of publication bias of country

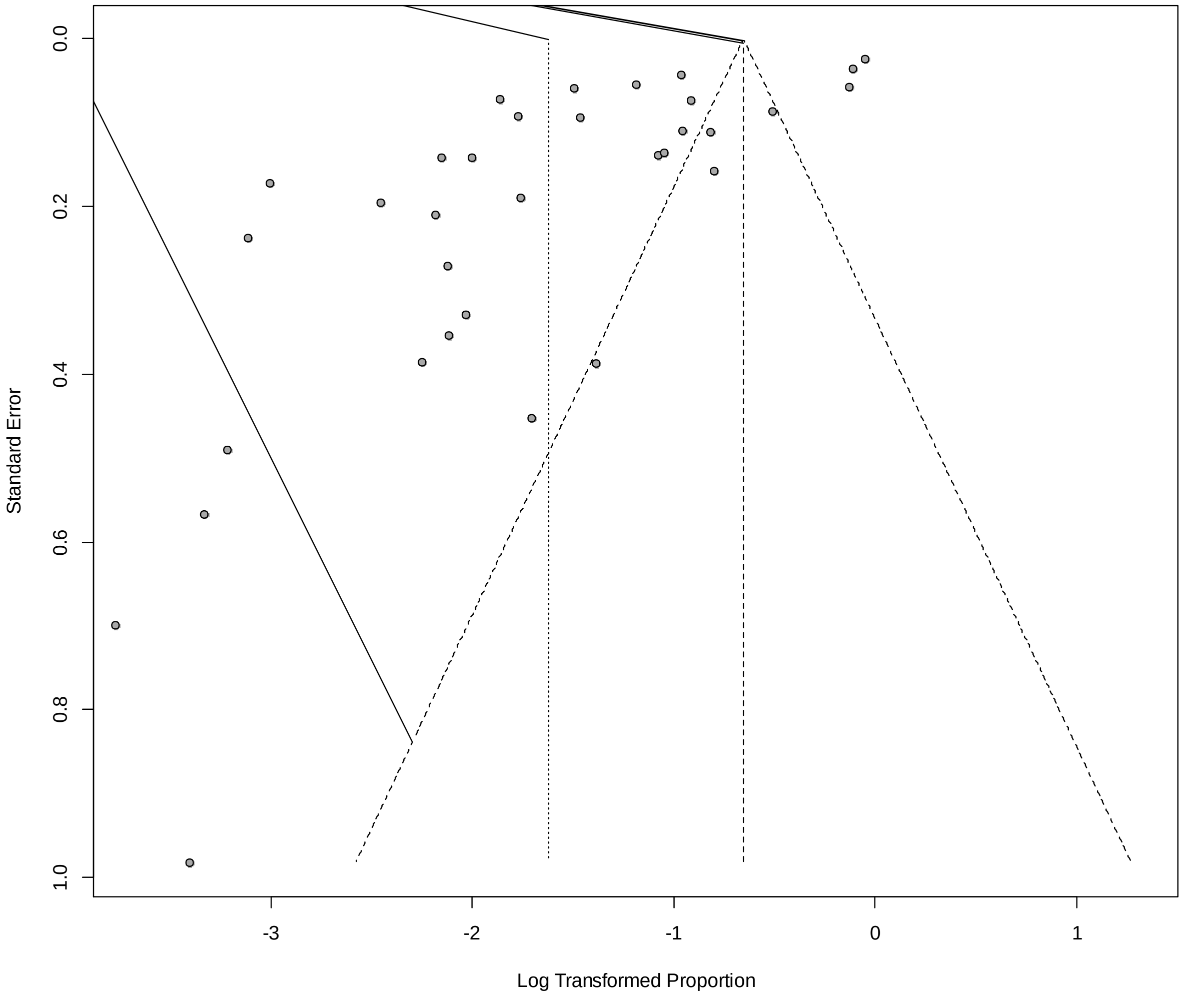

Figure S14. Funnel plot with pseudo 95% confidence limits intervals for the examination of publication bias of quality score
